# A Tool for Investigating Asthma and COPD Exacerbations: A Newly Manufactured and Well Characterised GMP Wild-Type Human Rhinovirus for Use in the Human Viral Challenge Model

**DOI:** 10.1371/journal.pone.0166113

**Published:** 2016-12-09

**Authors:** Daniel J. Fullen, Bryan Murray, Julie Mori, Andrew Catchpole, Daryl W. Borley, Edward J. Murray, Ganesh Balaratnam, Anthony Gilbert, Alex Mann, Fiona Hughes, Rob Lambkin-Williams

**Affiliations:** hVIVO Group PLC, Queen Mary BioEnterprises Innovation Centre, London, England, United Kingdom; University of North Carolina at Chapel Hill, UNITED STATES

## Abstract

**Background:**

Human Rhinovirus infection is an important precursor to asthma and chronic obstructive pulmonary disease exacerbations and the Human Viral Challenge model may provide a powerful tool in studying these and other chronic respiratory diseases. In this study we have reported the production and human characterisation of a new Wild-Type HRV-16 challenge virus produced specifically for this purpose.

**Methods and Stock Development:**

A HRV-16 isolate from an 18 year old experimentally infected healthy female volunteer (University of Virginia Children’s Hospital, USA) was obtained with appropriate medical history and consent. We manufactured a new HRV-16 stock by minimal passage in a WI-38 cell line under Good Manufacturing Practice conditions. Having first subjected the stock to rigorous adventitious agent testing and determining the virus suitability for human use, we conducted an initial safety and pathogenicity clinical study in adult volunteers in our dedicated clinical quarantine facility in London.

**Human Challenge and Conclusions:**

In this study we have demonstrated the new Wild-Type HRV-16 Challenge Virus to be both safe and pathogenic, causing an appropriate level of disease in experimentally inoculated healthy adult volunteers. Furthermore, by inoculating volunteers with a range of different inoculum titres, we have established the minimum inoculum titre required to achieve reproducible disease. We have demonstrated that although inoculation titres as low as 1 TCID_50_ can produce relatively high infection rates, the optimal titre for progression with future HRV challenge model development with this virus stock was 10 TCID_50_. Studies currently underway are evaluating the use of this virus as a challenge agent in asthmatics.

**Trial Registration:**

ClinicalTrials.gov NCT02522832

## Introduction

Human Rhinovirus (HRV) infections are frequently associated with the common cold and acute upper respiratory tract infection (URTI) in humans. Although often considered trivial, they are associated with significant economic implications as well as being an important predisposing factor in sinusitis, otitis media, bronchitis and primary pneumonia [1, 2]. Furthermore, HRV is known to cause considerable morbidity in certain at-risk groups such as infants, the elderly, the immunocompromised, and those with chronic respiratory disease like asthma, chronic obstructive pulmonary disease (COPD), and cystic fibrosis. At present, HRV is considered the number one cause of asthma exacerbations [3, 4]. Therefore, the use of HRV in a Human Viral Challenge (HVC) Model can be an extremely powerful tool, not just to study HRV infection and disease, but also to investigate the mechanisms of exacerbation in patients with chronic respiratory disease and to conduct efficacy studies for new therapies in these disease areas.

Asthma is defined as a heterogeneous disease, usually characterised by chronic airway inflammation, and defined by a history of respiratory symptoms such as wheeze, shortness of breath, chest tightness, and cough, which vary over time and in intensity, together with variable expiratory airflow limitation [5].

It is estimated that 300 million people worldwide are affected by asthma and annually approximately 250,000 people die from the disease. Many of the deaths are preventable and result from suboptimal long-term medical care and delay in obtaining help during severe exacerbations of the disease [6]. Prevention of symptom exacerbation, treatments for severe asthma, and curative therapies for mild to moderate asthma that do not result in a return to symptoms when the treatment stops are major unmet medical needs.

Human challenge studies with experimental HRV infection have been shown to produce infection in over 90% of serologically suitable subjects and result in a clinical syndrome that is comparable to that reported with natural colds [7, 8]. Symptoms usually appear within 24 hours and peak at 48–72 hours after inoculation, with virus shedding in infected subjects following a pattern similar to that of the symptoms. In recent times, several hundred inoculations of adult subjects have been reported and have established this as a safe and effective method in which to study HRV-related disease in both healthy and asthmatic subjects [7]. These studies provide a good knowledge base to develop the HRV experimental model and provide a controlled and effective tool to develop new therapies for the disease areas associated with HRV infection. New treatments for asthma and COPD are urgently needed and small animal models of asthma are poorly predictive of efficacy. Most drugs that are effective in animal models have failed in clinical trials, and drugs that might be effective would not be identified by these models. Models that more closely follow clinical features of human asthma are needed [9–14].

In order to develop the HRV HVC Model in new disease areas it is important to ensure availability of a safe, well-characterised challenge virus that has been rigorously tested for adventitious agents and produced in sufficient quantity to enable use of the same batch throughout the progression of the model development; this is particularly important in enabling the accumulation of directly comparable data as additional subjects are inoculated. Furthermore, to maximise the full utility of the HVC model and ensure that it is as safe and clinically relevant as possible in its mimicry of the natural infection, it is important to establish the minimal inoculation titre required to result in a consistent reproducible infection. Experimental HRV inoculations have previously used a wide variation of inoculum titres but have not established the minimum inoculation titre required.

We report the initial characterisation of a new Good Manufacturing Practice (GMP) grade stock of Wild-Type HRV-16 that we have produced with the aim of developing HRV-16 HVC models in patients with chronic respiratory diseases. We chose HRV-16 due to the extensive historical experience in the HVC Model with this virus [8–11, 15–22].

To maximise patient safety and the robustness of the data, and to prevent interference from community-acquired infections, we conducted the study in our purpose built quarantine unit. Our aims were to establish the safety of the virus in healthy adults before progressing to patient populations, and to determine the minimum inoculum titre required. Three viral titres were tested.

Human experimental HRV infection in mild asthmatic volunteers has been shown to be associated with augmented physiological and inflammatory responses, moderate reductions in peak expiratory flow (PEF) and forced expiratory volume in 1 second (FEV_1_), and increases in bronchial hyper-reactivity. Responses to HRV infection in asthmatic and healthy volunteers have determined that lower respiratory symptoms, lung function impairment, and bronchial hyper-reactivity were significantly greater in infected asthma subjects compared to healthy controls.[9–11, 20, 23]

Experimental HRV challenge provides a basis for decision-making in the development of asthma therapies, and the study of pharmacodynamics on experimental HRV challenge may allow the generation of testable hypotheses regarding mode of action, and new targets against which drugs can be developed.

## Methods

**Fig 1:**
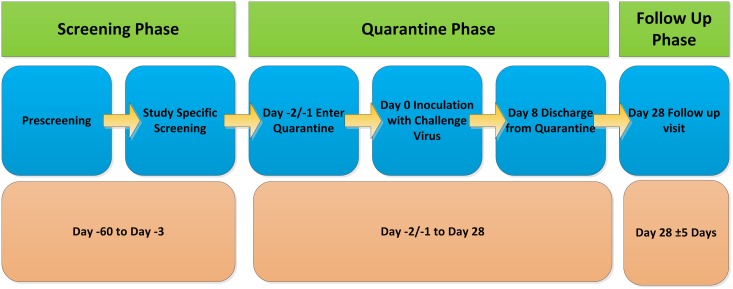
A Summary of the Clinical Trial Design.

The study was approved by Fulham National Research Ethics Service, London and the study was conducted in accordance with Good Clinical Practice and the Declaration of Helsinki 1996. We thank the committee for their constructive input. A summary of the clinical study design in accordance of the Consort principles is shown in Fig 1. All volunteers provided full written consent using a form approved by the committee.

### Challenge Virus Production

The HRV-16 virus was isolated from a nasal wash sample taken from an 18 year old experimentally infected healthy female volunteer (University of Virginia Children’s Hospital, USA), who developed symptoms consistent with a respiratory virus infection two days post HRV-16 infection. The decision was made in this instance to obtain a clinical isolate for the seed material as it enabled us to better anticipate the level of symptomology that could be expected based on the donors reported symptoms. It would be much more difficult predict the symptomology of a recombinant virus from sequence data alone. The donor underwent extensive health screening and was negative for Hepatitis B, C and Human Immunodeficiency Virus (HIV) types I and II and remained negative at a 5 year follow-up.

**Fig 2:**
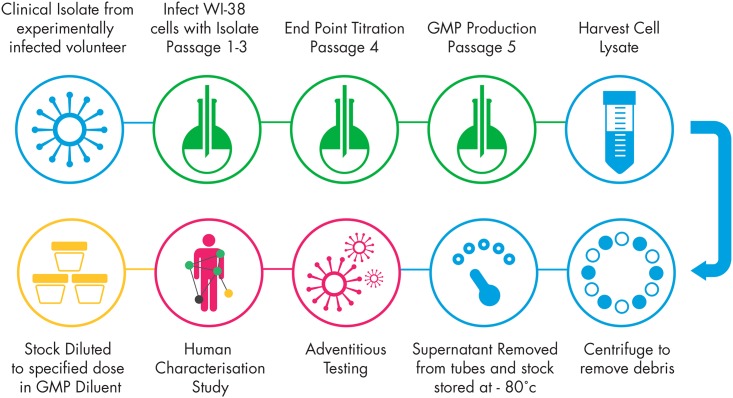
Summary of passages for production of the master virus seed stock.

WI-38 cells (ECACC; master cell bank produced to GMP standard) were inoculated with nasal lavage diluted at 10^−1^, 10^−2^ and 10^−3^ in infection medium and incubated at 33°C ± 2°C, 5% CO_2_ in T75 flasks and observed regularly for virus cytopathic effect (CPE). The virus was harvested when approximately 90% of cells showed CPE. Passage 1 was harvested from the flask infected with nasal lavage pre-diluted at 10^−1^ on day 3, underwent a freeze thaw and was clarified by centrifugation at 500 *g* for 10 mins. This was repeated for passage 2 in T225 flasks which were harvested from a 10^−1^ inoculation which was harvested on day 5 to produce passage 3. An end point titration was conducted on passage 3 which had a titre of 4.85 log10 TCID_50_/ml. The end point titration of passage 3 was harvested after 8 days in WI-38 cells (passage 4) was used as the seed material for the GMP production stock. Passage 5 was inoculated at a 10^−1^ dilution in T225 flasks and harvested 7 days post inoculation under GMP conditions (Bioreliance). The GMP stock underwent a freeze thaw, was clarified by centrifugation at 500 *g* for 10 min and the supernatants snap frozen in 1.1 ml aliquots for future use. The GMP virus stock had a titre of 5.68 log_10_ TCID_50_/ml in MRC-5 cells using the Spearman-Karber calculation. All reagents were selected to reduce the risk of contamination from bovine and porcine infectious agents in animal derived materials (e.g., foetal calf serum was of US or New Zealand origin; recombinant trypsin). The production process is shown in Fig 2.

Viral identity was determined by Sanger sequencing (Baseclear) and found to have 99% homology with HRV-16 Genbank sequence L24917 (S1 Fig). The GMP HRV-16 stock was screened for potential adventitious agents (viruses including, but not exclusively Hepatitis, A, B and C, HIV Types I and II and Human T-lympotropic virus (HTLV); mycoplasma; bacteria; fungi; and endotoxins). All results confirmed the suitability of the HRV-16 GMP stock for use in HVC studies. Human challenge inoculum vials were produced by diluting GMP HRV-16 virus stocks in a GMP diluent comprising of 25% sucrose in phosphate buffered saline (PBS) adjusted to a pH of 7.3.

### Microneutralisation Assay

Human sera was heat inactivated in a water bath at 56°C for 45 mins, 50 μl of sera was titrated across a flat bottom 96 well plate at two fold serial dilution in infection media consisting of Eagles minimum essential media (EMEM), 2% Foetal Calf Serum (FCS), 10 mM HEPES buffer, 2 mM L-Glutamine, 1% non-essential amino acids (NEAA), 10 mM MgCl2. The sera was incubated with 50 μl of HRV-16 at 3.3 Log_10_ TCID_50_/ml for 30 min at 30°C. 100 μl of MRC-5 cells at 1x 10^5^ cells/ml to each well and incubate for 5 days at 33°C in 5% CO_2_. The plates were observed after 5 days for signs of CPE, if less than 50% was observed relative to the virus only control a well was reported for neutralisation. The Reed-Muench calculation was used with replicate values to determine the value of neutralisation.

### Clinical Study Design

Study RVL-CS-002 was a randomized single blind study to characterize HRV-16 for use in future HVC studies (Fig 1). The primary objective was to determine a suitable infectious titre of HRV-16 that would yield infection in a high proportion of volunteers along with mild to moderate clinical symptoms in the majority of those infected. The study safety objective was to assess the safety of the infectious HRV-16 inoculum (i.e., incidence of virus challenge emergent adverse events (AEs) which were not consistent with a mild to moderate HRV-16 infection).

Healthy adult volunteers who were serosuitable for HRV-16 infection underwent study specific eligibility assessments which included physical examination, medical history, vital signs, electrocardiogram (ECG), spirometry, clinical chemistry, haematology, and coagulation. The viral challenge phase was conducted at the hVIVO quarantine facility in Whitechapel, London. Serosuitability was defined as the absence of detected antibodies in a volunteer’s serum to the challenge virus in the neutralisation assay. Screening of subjects for the study began 63 days prior to initiation and up to 10 days prior to commencement of the study. This was conducted to determine the subjects would be susceptible to the challenge virus prior to inoculation with the challenge virus. Subjects were admitted 48 hours before inoculation for repeat baseline eligibility assessments. Absence of a concurrent respiratory illness was confirmed by a negative Direct Fluorescence Antibody (DFA) assay LIGHT DIAGNOSTICS^™^ SimulFluor^®^ Respiratory Screen, Merckmillipore). Follow up visits we conducted 28 days after the end of the study.

Subjects were randomised to one of three virus titre groups, (1, 10, or 100 TCID_50_) and intranasally challenged whilst supine with a total of 1 mL (two inoculations of 250 μL per nostril) using a pipette. Subjects were randomly allocated to either quarantine groups A, B or C pre-inoculation via a randomisation code list. The randomisation code list was computer generated using a permuted block algorithm in a 1:1:1 ratio for the three quarantine groups. The subjects and investigator team were blinded to the allocation of Challenge Virus titre. The volunteers were inoculated with the Challenge Virus on Day 0 and remained in individual en-suite rooms in hVIVO’s quarantine facility until Day 8. Nasopharyngeal swabs were taken three times daily and nasal washes performed twice daily from Day 1 until discharge on Day 8; throat swabs were taken at specified times during quarantine. Subjects attended a follow up visit 28 days (+/- 5 days) after viral challenge, at which all baseline and safety assessments were repeated.

Various virological clinical, and safety laboratory assessments were performed during the study. Virus shedding was investigated in nasal washes, nasopharyngeal swabs and throat swabs by serial dilution and propagation in MRC-5 cells; and by reverse transcriptase quantitative polymerase chain reaction (RT-qPCR). Serology was performed at the Day 28 follow-up visit to determine the presence of HRV-16 neutralising antibodies.

Subjects self-completed hVIVO symptom diary cards three times daily to evaluate symptoms of upper respiratory tract (URT) illness (runny nose, stuffy nose, sore throat, sneezing); lower respiratory tract (LRT) illness (cough, shortness of breath), and systemic respiratory tract (SRT) illness (earache, malaise, headache, muscles and/or joint ache). Each of the 10 symptoms was rated from 0–3; 0 for ‘no symptoms, 1 (mild)–‘just noticeable’, 2 (moderate)–‘clearly bothersome but not affecting daily activities’, and 3 (severe)–‘quite bothersome with an effect on daily activities.

Mucus weights were assessed by subtracting the baseline weight of (unused) paper tissues from the final used weight. Pre-weighed paper tissues were distributed and collected on a daily basis during quarantine.

Daily directed physical examinations were performed to assess URT and LRT symptoms and correlate them with those reported by the subject. Vital signs, temperature, spirometry and ECG were recorded throughout the quarantine period.

### Virus Infection: Infectivity and Viral Shedding

Laboratory-confirmed HRV-16 infection was defined as the presence of viral shedding or seroconversion. Viral shedding was defined as at least one positive cell culture assay in any sample type (nasal wash, nasopharyngeal swab or throat swab), and/or at least two positive detections by qualitative polymerase chain reaction (qPCR) in any sample type (nasal wash, nasopharyngeal swab or throat swab) during the post-Viral Challenge quarantine period to the day of discharge. Seroconversion, as measured by serial dilution using a standard neutralisation assay, was defined as a four-fold or greater increase in serum neutralising antibody titre from baseline (between day -2 and day 28).

### Statistical Analysis

The primary endpoint was the area under the curve (AUC) of each HRV-16 virus titre load as determined by quantitative polymerase chain reaction (PCR). All subjects challenged with the HRV-16 virus were included in the analysis. The safety population and analysis subset refers to all randomised subjects receiving HRV-16 Challenge Virus inoculum. Statistical analysis was conducted using GraphPad Prism 6.

## Results

### Virus Manufacture

GMP grade HRV-16 was confirmed to be Wild-Type HRV-16 by Sanger sequencing. Results of sterility and adventitious testing of GMP grade HRV-16, which included the screening of more than 60 targets including Hepatitis, A, B and C, HIV Types I and II and HTLV, were negative to the limits of detection for the assays.

### Clinical Study

#### Subjects

Twenty-three subjects were invited to the quarantine phase of the study; six were excluded prior to inoculation on Day 0. Seventeen volunteers were enrolled, randomised and completed their follow up at Day 28 (+/-5 days). There were no substantial differences in baseline characteristics between the three virus dose groups (S1 Table).

#### Virology and infectivity

Fifteen (83%) of the 17 randomised subjects developed laboratory-confirmed infection (Table 1). In the 1 TCID_50_ group, four subjects (66.7%) had laboratory-confirmed infection; all four were positive by tissue culture and had at least two positive detections by qPCR of nasopharyngeal swab, nasal wash or throat swab. One of the laboratory-confirmed infected subjects did not seroconvert. Two subjects had no laboratory-confirmed infection post-viral challenge.

**Table 1:** Number of Subjects with Confirmed Infection by Laboratory Method Following Inoculation with HRV-16 (hVIVO).

Status	Viral titre group		
	1 TCID_50_	10 TCID_50_	100 TCID_50_
	N = 6	N = 6	N = 5
Positive tissue culture assay^a^	4	5	5
Positive qPCR^b^	4	5	5
Seroconversion	3	6	5
Laboratory-confirmed infection	4	6	5

^a^-By nasopharyngeal swab, nasal wash or throat swab

^b^-At least 2 positive detections in nasopharyngeal swab, nasal wash or throat swab.

Of the 17 subjects inoculated with HRV-16 (hVIVO), 15 developed laboratory-confirmed infection. All subjects (100%) in the 10 TCID_50_ and 100 TCID_50_ virus titre groups had laboratory confirmed HRV-16 (hVIVO) infection, compared to 4 (66.7%) of the 1 TCID_50_ virus titre group.

In the 10 TCID_50_ group, all six subjects had laboratory-confirmed infection. Five were positive by both tissue culture and qPCR (at least two positive detections of nasopharyngeal swab, nasal wash or throat swab). One subject did not have a positive tissue culture or qPCR but seroconverted and had laboratory confirmed HRV-16 infection.

In the 100 TCID_50_ group, all five subjects had laboratory-confirmed infection; all five were positive by tissue culture, and all had at least two positive detections by qPCR of nasopharyngeal swab, nasal wash or throat swab. All subjects in this group seroconverted. The peak in viral shedding occurred around Day 4 post viral challenge for each group.

Virus was not detected in the throat swabs of all the laboratory-confirmed infected volunteers and, when detected, the virus titres were low compared to the titres observed for nasal washes and nasopharyngeal swabs (data not shown). All laboratory-confirmed infected subjects had detectable virus by qPCR on discharge but were asymptomatic.

#### Seroconversion

Seroconversion was determined between Day -2 and Day 28. All subjects in the 10 TCID_50_ and 100 TCID_50_ groups seroconverted, whereas in the 1 TCID_50_ group only three subjects (50%) seroconverted. Two of the volunteers in the 1 TCID_50_ group did not seroconvert and did not shed virus.

### Illness Measures: Symptom Scores and Mucus Weights

#### Symptom scores

Subjects self-assessed 10 defined symptoms associated with HRV infection up to three times per day during quarantine (Day -2/-1 through to Day 8), with each defined symptom scored between 0 (none) and 3 (severe).

**Fig 3:**
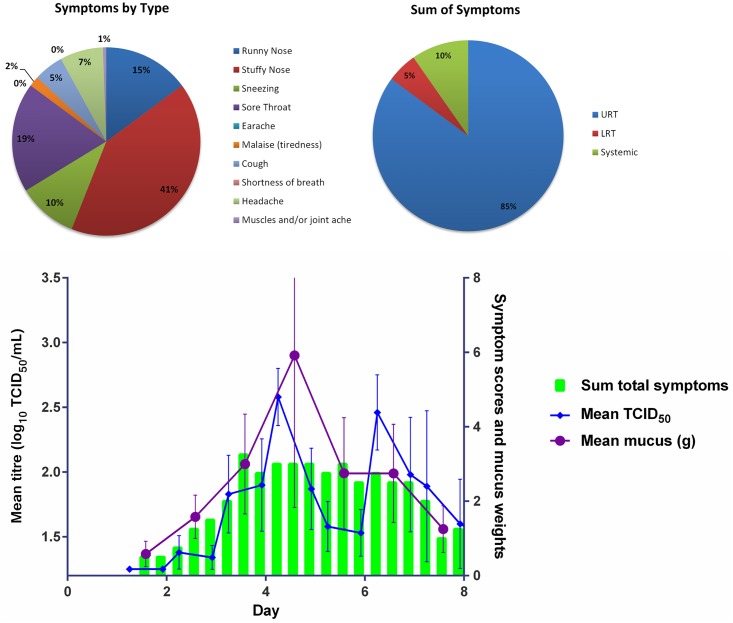
Mean Viral Load Titres (TCID_50_ Nasal Wash Samples), Mean Total Symptom Score and Mean Mucus Weight in Laboratory Confirmed Infected Volunteers from Day 1 to Day 8 in the 1 TCID50 Virus Titre Group. (Error Bars SEM)

**Fig 4:**
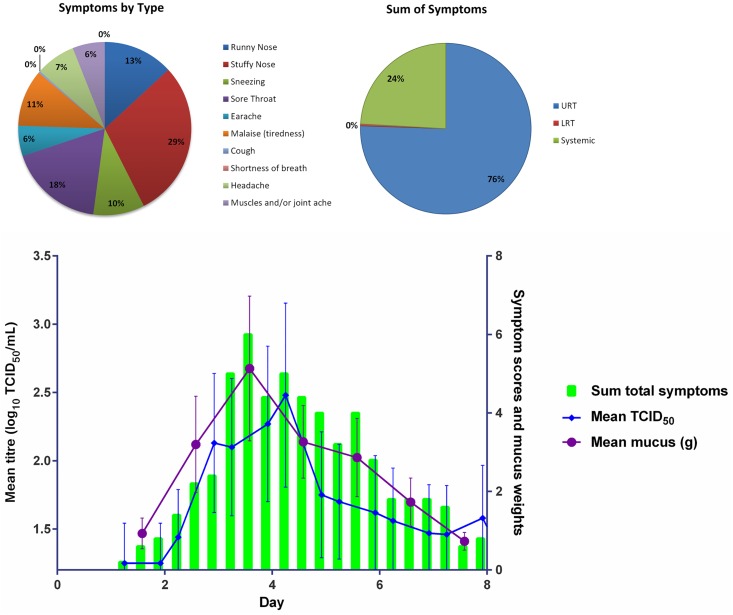
Mean Viral Load Titres (TCID_50_ Nasal Wash Samples), Mean Total Symptom Score and Mean mucus weight in Laboratory Confirmed Infected Volunteers from Day 1 to Day 8 in the 10 TCID50 Virus Titre Group. (Error Bars SEM)

**Fig 5:**
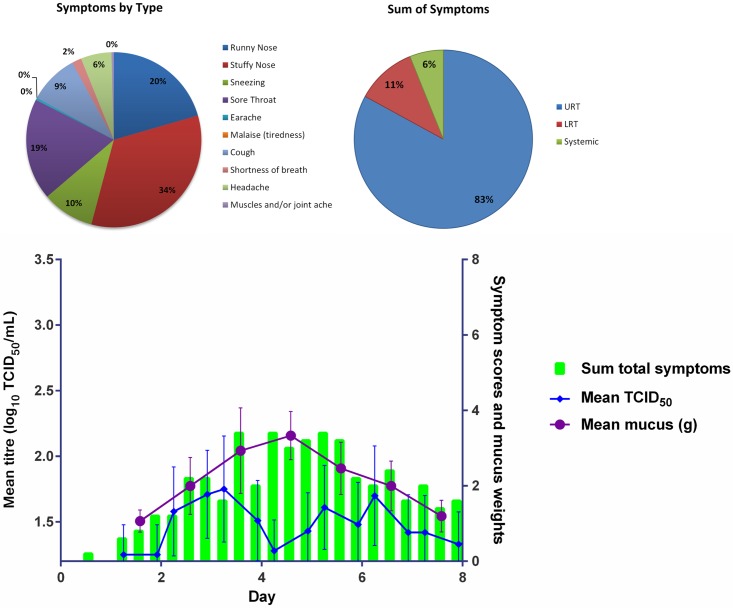
Mean Viral Load Titres (TCID_50_ Nasal Wash Samples), Mean Total Symptom Score and Mean Mucus Weight in Laboratory Confirmed Infected Volunteers from Day 1 to Day 8 in the 100 TCID_50_ Virus Titre Group. (Error Bars SEM)

Mean total symptom scores (any grade) over Days 1 to 8 for the laboratory-confirmed infected subjects for each of the different viral titre groups are shown in Figs 3, 4 and 5. The majority of the 17 subjects had symptoms consistent with an URTI (runny nose, stuffy nose, sore throat, sneezing). Sore throat, stuffy nose and runny nose were the main symptoms experienced by the subjects. Sore throat was a predominant early onset symptom (data not shown).

A summary of the time to peak, peak symptom score and duration of virus shedding symptom score for each of the three different virus titre inoculum groups is shown in Table 2. For all groups, symptoms peaked on Day 3 post viral challenge. Subjects in the 10 TCID_50_ group had the highest mean peak symptom score (9.8; SD:7.57), with the lowest mean peak symptom score in the 1 TCID_50_ group (8.5: SD = 5.57). Symptom duration was similar for the three groups (5.8 to 6.3 days), but longest for the 1 TCID_50_ group (6.3 days; SD:2.87).

**Table 2:** Time to Peak, Peak Value, and Duration of Symptoms of Any Grade from Day 0 (Post Challenge) To Day 8 in Laboratory-Confirmed Infected Subjects.

Symptoms	Viral titre group		
	1 TCID_50_	10 TCID_50_	100 TCID_50_
	N = 4	N = 6	N = 5
	Mean (SD)	Mean (SD)	Mean (SD)
Time to peak (days)	3.8 (0.96)	3.0 (0.63)	3.2 (1.92)
Peak value (score)	8.5 (5.57)	9.8 (7.57)	9.4 (5.46)
Duration (days)	6.3 (2.87)	5.8 (2.79)	6.0 (3.0)

SD, Standard deviation.

Time to Peak = Number of days from Day 0 until the peak is observed.

Peak value = the highest number of individual symptoms grade 1 or higher on a given Day between Day 0 (post challenge) to Day 8.

Duration = Time in days from the Day when a symptom of grade 1 or higher is first observed to the Day when a symptom of grade 1 or higher is last observed.

Table includes only volunteers with at least one symptom grade 1 or higher.

The occurrence of symptoms coincided with viral shedding. The symptom types and severity are in agreement with those reported during naturally-acquired infection [7]. Eight (47.0%) of the 17 subjects developed individual symptoms of Grade 2 or higher (Table 3). The 10 TCID_50_ group (66.7%) had incidences of any symptom, URT and SRT symptoms of Grade 2 or higher but no LRT symptoms of Grade 2 or higher. The 1 TCID_50_ group and 100 TCID_50_ group had incidence of any symptoms, URT and LRT of Grade 2 or higher but no incidences of SRT symptoms of Grade 2 or higher.

**Table 3:** Incidence Anytime of Symptoms of Grade 2 or Higher from Day 0 (post-challenge) to Day 8 in Inoculated Volunteers in Study RVL-CS-002.

Illness (with laboratory-confirmed infection)	Viral Titre Group		
	1 TCID_50_	10 TCID_50_	100 TCID_50_
	N = 6 (%)	N = 6 (%)	N = 5 (%)
Any symptom—Grade 2 or higher	2 (33.3)	4 (66.7)	2 (40.0)
URT- Grade 2 or higher	2 (33.3)	4 (66.7)	2 (40.0)
LRT- Grade 2 or higher	1 (16.7)	0	1 (20.0)
SRT—Grade 2 or higher	0	2 (33.3)	0

URT, Upper Respiratory Tract symptoms based any 1 of the following on 2 consecutive days, at least 1 Day of which must attain Grade 2 severity, or if any of the following attain Grade 3 severity once (in conjunction with the physician’s directed physical examination) to correlate findings: rhinorrhea (runny nose), nasal congestion (stuffy nose), sneezing, sore throat.

LRT, Lower Respiratory Tract symptoms based any 1 of the following on 2 consecutive days, at least 1 Day of which must attain Grade 2 severity, or if any of the following attain Grade 3 severity once: self-reported symptom of cough and/or physician findings of new wheezes, râles, rhonchi or other lower respiratory tract signs.

SRT, Systemic Respiratory Tract symptoms based on any 1 of the following on 2 consecutive days, at least 1 Day of which must attain Grade 2 severity, or if any of the following attain Grade 3 severity once: headache persisting for >1 hour, myalgia and/or arthralgia, general malaise, chilliness/fever.

#### Mucus weights

**Fig 6:**
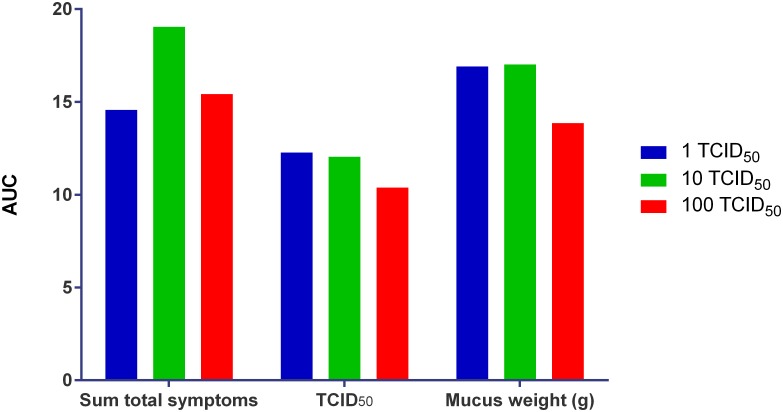
AUC of Total Symptoms, Viral Shedding (TCID_50_ Nasal Wash Samples) and Mucus Weight.

Both the mean and median mucus weights were greatest for the 100 TCID_50_ group, except on Day 3 where the median equalled that of the 10 TCID_50_ group (4.31 g). The median mucus weight peaked on Day 5 (5.77 g [SD: 5.57]) for the 10 TCID_50_ group, on Day 5 for the 1 TCID_50_ and 100 TCID_50_ groups, and on Day 4 for the 10 TCID_50_ group. The AUCs of TCID_50_ of nasal wash samples, total symptoms, and mucus weights for each viral titre group are shown in Fig 6.

#### Virus expected and adverse events

A Virus Expected Event (VEE) is that which would be recorded in a symptom diary card or is consistent with a common cold-like illness. Sixteen subjects developed VEEs (Table 4). VEEs not consistent with a mild to moderate HRV-16 infection were reported by only one subject, and were considered to be related to the study procedures, the quarantine environment, and subject’s activities following the quarantine phase of the study.

**Table 4:** Summary of Subjects with Virus Expected Events in the Safety Analysis and Laboratory-Confirmed Infection Sets.

Analysis set	Virus titre group						Total	
	1 TCID_50_		10 TCID_50_		100 TCID_50_			
	No. of subjects (%)	No. of events	No. of subjects (%)	No. of events	No. of subjects (%)	No. of events	No. of subjects (%)	No. of events
Safety Analysis Set	6 (100.0)	20	5 (83.3)	17	5 (100.0)	17	16 (94.1)	54
Laboratory-confirmed infected subjects	4 (100.0)	12	5 (83.3)	17	5 (100)	17	14 (93.3)	46

Three subjects had abnormal spirometry during the study, which in some cases was considered possibly related to the Challenge Virus although the AE resolved and no action was required. One serious adverse event (SAE) was reported in the 1 TCID_50_ group. The SAE was a Grade 4 self-limiting raised creatine phosphokinase considered to be of medical importance, but not considered to be related to the Challenge Virus, study procedures, concomitant medications or study assessments. No action was taken and the SAE resolved.

## Discussion

The primary objective of the study was to determine a suitable infectious titre of Wild-Type HRV-16 stock for use as a Challenge Virus in future HVC studies and specifically for investigations into the causes of viral exacerbation of airways disease, such as asthma and COPD. Post GMP production this was the first time this virus had been assessed *in vivo* with an n of 6 per group, hence its exploratory nature. This study design and size is typical of a first in man human challenge study with its small sample size in order to minimise the risk to healthy volunteers [24–28].

We found that across all dose groups the peak in viral shedding occurred around Day 4 post challenge and the peak in mean mucus weights around Day 4–5 post challenge. Although the peak titres around Day 4 post challenge were comparable for the 1 TCID_50_ and 10 TCID_50_ groups, the sum of the total symptoms for the 10 TCID_50_ inoculum group was the highest of the three challenge groups used.

The main symptoms reported across all dose groups were stuffy nose (29–41%), sore throat (18–19%), and runny nose (13–20%), all of which are classic common cold symptoms. In all dose groups the vast majority of the symptoms were associated with the URT (76–85%). In the 1 TCID_50_ and 10 TCID_50_ dose groups the SRT symptoms were the second most frequently recorded symptoms, only in the 100 TCID_50_ dose group were LRT symptoms more frequently reported than SRT symptoms.

Interestingly, while the 100 TCID_50_ dose group had the lowest overall viral shedding, this was the only group to display a higher proportion of LRT symptoms than SRT symptoms. The low levels of virus shedding identified in the 100 TCID_50_ group may have been a consequence of the production of defective interfering virus due to a high multiplicity of infection in which interfering particles limit virus replication. This does not however explain the increased levels of LRT symptoms which could be due to priming of the immune system due to the exposure to an increased antigenic load. This could however, be a result of natural variation given the small sample sizes. As we build on this body of data in the future with increasing n numbers in our dedicated human challenge centre, our understanding of the progression of rhinoviral infection and the underlying causes of its pathology will become much clearer.

The 10 TCID_50_ titre was shown to be safe and pathogenic and was considered the preferential titre across all parameters. It had an equal laboratory-confirmed infection rate to 100 TCID_50_ virus titre group, both demonstrating a 100% laboratory-confirmed infection rate. Overall the 10 TCID_50_ virus titre produced the highest peak values and overall longest duration for viral shedding, which are the key elements in determining the size of the AUC for viral shedding (Table 2). The 10 TCID_50_ viral titre group therefore has the greatest potential to demonstrate a decrease in viral AUC by an investigational medicinal product (IMP).

This study shows that the hVIVO Wild-Type HRV-16 stock consistently produces disease in inoculated healthy volunteers with symptomology comparable to that of the natural infection. In addition we have demonstrated for the first time that, even though optimal infection rates and disease profiles are obtained with an inoculation titre of 10 TCID_50_, high infection rates can still be achieved with an inoculum titre of 1 TCID_50_. We have, therefore, established the minimum titre required for a reproducible infection in the HVC model with our HRV-16 batch, thus enabling closer mimicry of the natural infection than may be achieved using the higher inoculation titres previously reported by others. We anticipate this will provide a more sensitive model for the assessment of novel IMPs and discovery of key biomarkers. This may be particularly important in using the HVC model to investigate sensitive biomarkers of infection and disease progression, where the expression profile obtained may otherwise be inadvertently altered, either temporally or in magnitude, due to significantly higher inoculum titres being used than may be involved in natural infection.

## Conclusion

Having established that our new Wild-Type HRV-16 stock is both safe and pathogenic given the disease profile that it induces, we are continuing with the development of the HRV-16 HVC models. However, due to the limited number of individuals included in each study group, we have limited power to make formal statistical comparisons between the groups. Studies to increase the body of safety and pathogenicity data in healthy adults at the optimal titre based on the data presented are ongoing, and will enable us to progress into asthmatic and COPD patient populations.

Our HRV-16 stock has been produced in sufficient quantity to enable the same batch to be used throughout the development of the different planned models, thus building up an important body of safety and efficacy data.

## Supporting Information

S1 Fig Sequence Analysis of GMP HRV-16 Challenge Virus. (DOCX)

S1 Table Subject Baseline Demographics by Titre Randomisation at Screening. (DOCX)

S2 Table Summary of Viral Shedding Detection by Swab. (DOCX)

S3 Table Mean Viral Load Titres (Log Values) by Nasopharyngeal Swab qPCR in Laboratory Confirmed Infected Subjects from Days 1–8. (DOCX)

S4 Table CONSORT 2010 Checklist. (DOC)

S1 Text Clinical Study Protocol. (PDF)
