# Supplementary material for: A Tool for Investigating Asthma and COPD Exacerbations: A Newly Manufactured and Well Characterised GMP Wild-Type Human Rhinovirus for Use in the Human Viral Challenge Model
Source: PLoS One. 2016 Dec 9;11(12):e0166113. doi: 10.1371/journal.pone.0166113 (PMC5147828; doi:10.1371/journal.pone.0166113)
Supplement: S1 Fig — (DOCX) [file pone.0166113.s001.docx]

**S1 Fig: Sequence Analysis of GMP HRV-16 Challenge Virus**

GMP HRV16 RVL polyprotein sequence has 99% identity with GenBank L24917 HRV-16 polyprotein sequence and 94-99% identity with more recent oronasal isolates. We conclude that the GMP HRV RVL sequence is 100% conserved with three other HRV-16 sequences and shows marginal higher homology with the 1995 sequence compared to more recent isolates.

The table below shows a more detailed sequence analysis of GMP HRV16 RLV relative to the consensus sequence (CON) in multiple sequence alignments. A number of differences can be found with the majority also found in the L24917 HRV16 polyprotein sequence. There are two changes specific to GMP HRV16-RVL namely; V635L and K1522R. These amino acid changes map to VP1 and 3C respectively (see below) and are very conservative regarding amino functionality.

| Position | CON seq | GMP seq | Unique to GMP? | Gene | GMP seq also found in … |
| --- | --- | --- | --- | --- | --- |
| 635 | V | L | YES | VP4 (capsid) |  |
| 684 | T | A | NO | VP4 (capsid) | AAA69862 |
| 836 | T | S | NO | VP4 (capsid) | AAA69862 |
| 890 | V | I | NO | 2A | AAA69862 |
| 895 | N | S | NO | 2A | AAA69862 |
| 1196 | N | T | NO | 2C (helicase) | AAA69862 |
| 1522 | K | R | YES | 3C (cys protease) |  |
| 1544 | V | I | NO | 3C (cys protease) | AAA69862 |
| 1744 | E | K | NO | 3D (RdRp) | AAA69862 |
| 2178 | A | T | NO | 3D (RdRp) | AAA69862 |
