# Supplementary material for: A Tool for Investigating Asthma and COPD Exacerbations: A Newly Manufactured and Well Characterised GMP Wild-Type Human Rhinovirus for Use in the Human Viral Challenge Model
Source: PLoS One. 2016 Dec 9;11(12):e0166113. doi: 10.1371/journal.pone.0166113 (PMC5147828; doi:10.1371/journal.pone.0166113)
Supplement: S1 Table — (DOCX) [file pone.0166113.s002.docx]

**S1 Table: Subject Baseline Demographics by Titre Randomisation Group at Screening**

| Group | ID | Age | Sex | Ethnicity | Weight (kg) | BMI  (kg/m2) |
| --- | --- | --- | --- | --- | --- | --- |
| 1 TCID_50_ | RVL001 | 23 | M | White and Asian | 75.7 | 25.18 |
|  | RVL005 | 21 | M | White: British | 77.4 | 23.34 |
|  | RVL008 | 20 | M | White: British | 92.8 | 26.97 |
|  | RVL011 | 20 | M | Black or Black British: African | 86.0 | 25.40 |
|  | RVL014 | 23 | M | White: British | 55.5 | 18.44 |
|  | RVL016 | 21 | M | White: British | 66.1 | 22.47 |
| 10 TCID_50_ | RVL003 | 19 | M | White: British | 71.0 | 20.09 |
|  | RVL004 | 25 | M | White: British | 80.1 | 27.65 |
|  | RVL009 | 20 | M | White: British | 68.2 | 18.69 |
|  | RVL012 | 34 | M | White: British | 96.1 | 25.25 |
|  | RVL015 | 22 | M | White: British | 71.5 | 23.51 |
|  | RVL017 | 35 | M | White: British | 83.3 | 24.18 |
| 100 TCID_50_ | RVL002 | 28 | M | White: British | 77.5 | 27.36 |
|  | RVL006 | 23 | M | Black or Black British: African | 77.2 | 24.09 |
|  | RVL007 | 29 | M | White: British | 65.0 | 21.97 |
|  | RVL010 | 26 | M | White: British | 65.7 | 22.89 |
|  | RVL013 | 22 | M | White: British | 92.3 | 27.56 |
