# Supplementary material for: A Tool for Investigating Asthma and COPD Exacerbations: A Newly Manufactured and Well Characterised GMP Wild-Type Human Rhinovirus for Use in the Human Viral Challenge Model
Source: PLoS One. 2016 Dec 9;11(12):e0166113. doi: 10.1371/journal.pone.0166113 (PMC5147828; doi:10.1371/journal.pone.0166113)
Supplement: S2 Table — (DOCX) [file pone.0166113.s003.docx]

**S2 Table: Summary of Viral Shedding Detection by Swab Sample for All Subjects**

| Group | Subject | Nasal swab qPCR | | Nasal Lavage | | Throat swab | |
| --- | --- | --- | --- | --- | --- | --- | --- |
|  |  | qPCR | Tissue culture | qPCR | Tissue culture | qPCR | Tissue culture |
|  |  | Virus detected? Yes/ No | | | | | |
| 1 TCID_50_ | RVL001 | No | No | No | No | No | No |
|  | RVL005 | Yes | Yes | Yes | Yes | Yes | No |
|  | RVL008 | Yes | Yes | Yes | Yes | Yes | No |
|  | RVL011 | No | No | No | No | No | No |
|  | RVL014 | Yes | Yes | Yes | Yes | No | No |
|  | RVL016 | Yes | Yes | Yes | Yes | No | No |
| 10 TCID_50_ | RVL003 | Yes | Yes | Yes | Yes | Yes | No |
|  | RVL004 | Yes | Yes | Yes | Yes | No | Yes |
|  | RVL009 | Yes | Yes | Yes | Yes | No | No |
|  | RVL012 | Yes | Yes | Yes | Yes | Yes | No |
|  | RVL015 | Yes | Yes | Yes | Yes | No | No |
|  | RVL017 | No | No | No | No | No | No |
| 100 TCID_50_ | RVL002 | Yes | Yes | Yes | Yes | No | No |
|  | RVL006 | Yes | Yes | Yes | Yes | No | No |
|  | RVL007 | Yes | Yes | Yes | Yes | No | No |
|  | RVL010 | Yes | Yes | Yes | Yes | No | No |
|  | RVL013 | Yes | Yes | Yes | Yes | No | No |
