# Supplementary material for: A Tool for Investigating Asthma and COPD Exacerbations: A Newly Manufactured and Well Characterised GMP Wild-Type Human Rhinovirus for Use in the Human Viral Challenge Model
Source: PLoS One. 2016 Dec 9;11(12):e0166113. doi: 10.1371/journal.pone.0166113 (PMC5147828; doi:10.1371/journal.pone.0166113)
Supplement: S3 Table — (DOCX) [file pone.0166113.s004.docx]

**S3 Table: Mean Viral Load Titres (Log Values) by Nasopharyngeal Swab qPCR in Laboratory-confirmed Infected Subjects from Days 1-8**

| Group | Subject No | Peak titres (log_10_TCID_50_/mL) | | | |
| --- | --- | --- | --- | --- | --- |
|  |  | Nasal wash qPCR | Nasal wash culture | Nasopharyngeal swab qPCR | Nasopharyngeal swab culture |
| 1 TCID_50_ | RVL001 | Negative | Negative | Negative | Negative |
| 100 TCID_50_ | RVL002 | 1.82 | 2.00 | 0.35 | 1.62 |
| 10 TCID_50_ | RVL003 | 2.67 | 2.62 | 2.74 | 3.63 |
| 10 TCID_50_ | RVL004 | 3.45 | 3.52 | 1.96 | 2.75 |
| 1 TCID_50_ | RVL005 | 3.07 | 3.63 | 2.07 | 3.37 |
| 100 TCID_50_ | RVL006 | 1.76 | 2.62 | 0.96 | 2.17 |
| 100 TCID_50_ | RVL007 | 1.71 | 2.48 | 1.39 | 2.20 |
| 1 TCID_50_ | RVL008 | 2.63 | 3.00 | 1.80 | 2.37 |
| 10 TCID_50_ | RVL009 | 1.99 | 3.12 | 1.78 | 2.37 |
| 100 TCID_50_ | RVL010 | 1.65 | 1.87 | 0.37 | Detected |
| 1 TCID_50_ | RVL011 | Negative | Negative | Negative | Negative |
| 10 TCID_50_ | RVL012 | 1.98 | 2.13 | 0.85 | 2.12 |
| 100 TCID_50_ | RVL013 | 1.68 | 2.37 | 1.31 | 2.12 |
| 1 TCID_50_ | RVL014 | 2.04 | 2.25 | 1.92 | 2.85 |
| 10 TCID_50_ | RVL015 | 2.50 | 3.25 | 1.12 | 2.37 |
| 1 TCID_50_ | RVL016 | 1.54 | 2.39 | 1.32 | 1.75 |
| 10 TCID_50_ | RVL017 | 2.80 | Negative | Negative | Negative |
