# Supplementary material for: A Tool for Investigating Asthma and COPD Exacerbations: A Newly Manufactured and Well Characterised GMP Wild-Type Human Rhinovirus for Use in the Human Viral Challenge Model
Source: PLoS One. 2016 Dec 9;11(12):e0166113. doi: 10.1371/journal.pone.0166113 (PMC5147828; doi:10.1371/journal.pone.0166113)
Supplement: S1 Text — (PDF) [file pone.0166113.s006.pdf]

RETROSCREEN VIROLOGY
CONQUERING VIRAL DISEASE

CLINICAL STUDY PROTOCOL

Study Number: RVL-CS-002

A Randomised Single Blind Study to Characterise a Human Rhinovirus Type 16 for Use in Future Human Viral Challenge Studies

Version:	Final v2.5: 26th February 2013
Sponsor:	Retroscreen Virology Limited<br>Queen Mary BioEnterprises Innovation Centre<br>42 New Road<br>London E1 2AX UK
Chief Investigator :	Dr Bryan Murray<br>Retroscreen Virology Limited<br>Queen Mary BioEnterprises Innovation Centre<br>42 New Road<br>London E1 2AX UK
Study sites	Retroscreen Virology Limited<br>Queen Mary BioEnterprises Innovation Centre<br>42 New Road<br>London<br>E1 2AX. UK<br><br>Retroscreen Virology Limited<br>Angel House<br>24 Station Road<br>Ely<br>Cambridgeshire<br>CB7 4BS. UK
REC Reference	12/LO/1777

1 STUDY PERSONNEL CONTACT LIST

RVL SPONSOR	Ms Kym Denny<br>Retroscreen Virology Limited<br>Queen Mary BioEnterprises Innovation Centre<br>42 New Rd<br>London<br>E1 2AX<br>T: +44 (0) 20 7756 1367<br>E-mail: k.denny@retroscreen.com
CHIEF INVESTIGATOR OR DELEGATE	Dr Bryan Murray<br>Retroscreen Virology Limited<br>Queen Mary BioEnterprises Innovation Centre<br>42 New Rd<br>London<br>E1 2AX<br>T: +44 (0) 20 7756 1401<br>E-mail b.murray@retroscreen.com
MEDICAL EXPERT	Dr Ganesh Balaratnam<br>Retroscreen Virology Limited<br>Queen Mary BioEnterprises Innovation Centre<br>42 New Rd<br>London<br>E1 2AX<br>T: +44 (0) 20 7756 1300<br>E-mail: g.balaratnam@retroscreen.com
CRO STATISTICIAN	Mark Baillet<br>Managing Director and Consultant Statistician<br>S-Cubed Biometrics Ltd<br>99 Milton Park<br>Abingdon<br>Oxfordshire<br>OX14 4RY<br>T: +44 (0) 1235841 527<br>M: +44 (0) 7794085415<br>E: mb@s-cubed.co.uk
STUDY SITE 1	Retroscreen Virology Limited<br>Queen Mary BioEnterprises Innovation Centre<br>42 New Rd<br>London<br>E1 2AX
STUDY SITE 2	Retroscreen Virology Limited<br>Angel House<br>24 Station Road<br>Ely<br>Cambridgeshire<br>CB7 4BS<br>Tel: 02030210178

LABORATORY	Retroscreen Virology Limited<br>Queen Mary BioEnterprises Innovation Centre<br>42 New Rd<br>London<br>E1 2AX<br><br>The Doctors' Laboratory<br>60 Whitfield Street<br>London<br>WC1T 4EU<br><br>Further Laboratory details can be found in the Site File Contacts list and the Analytical Plan

This study will be conducted in accordance with the standards of Good Clinical Practice (GCP); the Declaration of Helsinki (1996) and the study protocol.

Table of Contents

1 STUDY PERSONNEL CONTACT LIST ... 2

2 SIGNATURE PAGE ... 8

3 PROTOCOL SYNOPSIS ... 9

4 ABBREVIATIONS AND DEFINITIONS ... 13

5 INTRODUCTION ... 17
5.1 The Challenge Virus: Human Rhinovirus (HRV) ... 17
5.2 Rationale for Study ... 18
5.3 Potential Risks ... 18

6 STUDY OBJECTIVES AND ENDPOINTS ... 20
6.1 Primary Objective ... 20
6.1.1 Primary Endpoint ... 20
6.2 Safety Objective ... 20
6.2.1 Safety Endpoint ... 20
6.3 Secondary Objective(s) ... 20
6.3.1 Secondary Endpoints ... 20

7 STUDY POPULATION ... 22
7.1 Inclusion Criteria ... 22
7.2 Exclusion Criteria ... 23
7.3 Subject Numbering ... 25
7.4 Subject Withdrawal Criteria ... 25
7.5 Discontinuation of Subject Participation ... 25
7.6 Discontinuation of the Study ... 26

8 STUDY DESIGN ... 27
8.1 Summary of Study Design ... 27
8.1.1 Screening ... 27
8.1.2 Quarantine and Challenge Phase ... 27
8.1.3 Follow-up Visit ... 28
8.2 Duration of the Study ... 28

9 CHALLENGE VIRUS ... 29
9.1 Production ... 29
9.2 Storage and Accountability ... 29
9.3 Administration ... 29
9.4 Unused Challenge Virus ... 29

10 STUDY RESTRICTIONS ... 30
10.1 Concomitant Medications ... 30
10.2 Other Restrictions ... 30

11 STUDY PROCEDURES ... 31
11.1 Time and Events Schedule ... 31
11.2 Screening Visit (Day -60 to Day -3) ... 34
11.3 Day -2 and Day -1 ... 34
11.4 Viral Challenge (Day 0) ... 35
11.4.1 Pre-challenge ... 35
11.5 Day 0 ... 36
11.5.1 Viral Challenge ... 36
11.5.2 Post-challenge ... 36

11.6 Quarantine Study Days 1 to 7 36
11.6.1 Exceptions 37
11.7 Discharge from Quarantine (Day 8) 37
11.8 Withdrawal Visit (Day 0 to 7) 38
11.9 Study Follow-up Visit (Day 28 ± 5 days) 38
11.10 Subject Discontinuation (Lost to Follow-up) 39

12 DEFINITIONS OF ILLNESS AND INFECTION 40
12.1 Upper Respiratory Tract Infection 40
12.2 Lower Respiratory Tract Infection 40
12.3 Systemic Respiratory Tract Illness 40
12.4 Laboratory-Confirmed HRV-16 (RVL) Illness 40
12.4.1 Laboratory-Confirmed HRV-16 (RVL) Infection 41
12.5 Definition of Viral Shedding 41
12.6 Definition of Seroconversion 41

13 CLINICAL ASSESSMENTS 42
13.1 Prior and Concomitant Medications 42
13.2 Height, Weight, BMI and Body Fat 42
13.3 Alcohol Breath Test 42
13.4 Physical Examination 42
13.4.1 Complete Physical Examination 42
13.4.2 Directed Physical Examination 43
13.5 Vital Signs 43
13.6 Temperature 44
13.7 12-Lead Electrocardiogram 44
13.8 Spirometry 44
13.9 Tissue Count and Mucus Weight 45
13.10 Symptom Diary Card 45
13.11 Adverse Events 45

14 LABORATORY SAFETY ASSESSMENTS 47
14.1 Blood 47
14.1.1 Haematology and Biochemistry 47
14.1.2 Coagulation 48
14.1.3 Cardiac Enzymes 48
14.1.4 HIV, Hepatitis B and C 48
14.1.5 Blood Glucose 48
14.1.6 Pregnancy Test 49
14.2 Urine 49
14.2.1 Urinalysis 49
14.2.2 Class A Drugs and Cotinine Screen 49
14.2.3 Pregnancy test 49

15 SAMPLES FOR VIRAL INFECTIVITY 50
15.1 Throat Swab 50
15.2 Nasal Swab 50
15.3 Nasal Brush 50
15.4 Nasal Scrape 50
15.5 Nasal Wash 50
15.6 Exhaled Breath Condensate 50

16 SAMPLE ANALYSIS 52
16.1 HRV-16 (RVL) Neutralisation Assay 52
16.2 Direct Fluorescence Antibody – (DFA) - Respiratory Viral Panel 52
16.3 Quantitative Polymerase Chain Reaction (qPCR) 52
16.4 Inflammatory Biomarkers (PBMC) 52

16.4.1 Humoral Immunity 52
16.4.2 Cell-Mediated Immunity 52
16.4.3 Viral RNA 52
16.4.4 Tissue Culture Infective Titre ($TCID_{50}$) 53

17 ADVERSE EVENTS 54

17.1 Serious Adverse Events 54
17.2 Adverse Drug Reaction 55
17.3 Clinical Laboratory Abnormalities and Other Abnormal Assessments 55
17.4 Recording of Adverse Events and Serious Adverse Events 56
17.4.1 Description 56
17.4.2 Onset and End 56
17.4.3 Intensity and Severity 56
17.4.4 Seriousness 57
17.4.5 Relationship or Causality 57
17.4.6 Outcome 57
17.4.7 Action Taken 58
17.4.8 Follow-up 58
17.4.9 Pregnancy 58
17.5 Reporting SAEs 59
17.5.1 Research Ethics Committee 60
17.6 Post-Study Obligations 60

18 STATISTICAL METHODS AND PLANNED ANALYSES 61

18.1 Study Populations 61
18.2 Data Management 61
18.3 Data Coding 62
18.4 Sample Size 62
18.5 Interim Analysis 63
18.6 Randomisation 63
18.7 Statistical and Analytical Plan 63
18.7.1 Subject Accountability 63
18.7.2 Protocol Deviations 64
18.7.3 Subgroup Analysis 64
18.7.4 Demographic and Baseline Characteristics 64
18.7.5 Virulence and Pathogenicity Analysis 64
18.7.6 Safety Analysis 64

19 STUDY MANAGEMENT AND RESPONSIBILITIES 67

19.1 Ethics Review 67
19.2 Regulatory Approval and Good Clinical Practice 67
19.3 Protocol Deviations 67
19.3.1 Serious Breach 67
19.4 Protocol Amendments 67
19.5 Discontinuation of the Study 68
19.6 Study Records Retention and Direct Access to Source Documents 68
19.7 Sponsor Responsibilities 68
19.8 Monitoring and Auditing 68
19.9 CI or Delegate Responsibilities 69
19.9.1 Ethical Considerations 69
19.9.2 Informed Consent 69
19.10 Laboratory Certification and Normal Values 70
19.11 Delegation of CI or delegate Responsibilities 70
19.12 Information for Subject’s General Practitioner 70
19.13 Compensation and Expenses 70
19.14 Non-Protocol Research 70

19.15 Liability and Insurance 70
19.16 Quality Assurance 70
19.17 Study Termination 71

20 DISCLOSURE OF DATA 72
20.1 Subject Confidentiality 72
20.2 Sponsor Confidentiality 72
20.3 Publication Policy 72
20.4 Study Documentation 72
20.4.1 Source Documents 72

21 REFERENCES 73

22 APPENDICES 74
Appendix 1: Study Approved Contraceptive Methods 75
Appendix 2: Excluded Medical and Psychiatric Conditions 76
Appendix 3: RVL Symptom Diary Card 78

LIST OF IN-TEXT TABLES

Table 1: Challenge Virus Titre for Quarantine Groups 28
Table 2: Time and Events Schedule 32
Table 3: Classification of AE Causality 57
Table 4: Classification of AE Outcome 58

LIST OF IN-TEXT FIGURES

Figure 1: Study Design for Subjects 27

2 SIGNATURE PAGE

SPONSOR'S AUTHORISATION

A designated professional representative of the Sponsor will verify adherence to the protocol and the accurate and complete recording of data in the source documents.

PROTOCOL AGREED BY SPONSOR REPRESENTATIVE

SPONSOR REPRESENTATIVE
SIGN ______________ DATE 04 / MAR / 2013
~~MS KYM DENNY~~ GARETH RIDGE

PROTOCOL AGREED BY CHIEF INVESTIGATOR OR DELEGATE

I have read and agree this protocol. I am aware of my responsibilities as a CI or delegate under the guidelines of ICH GCP, the Declaration of Helsinki (1996) and the study protocol. I agree to conduct the study according to these guidelines and to appropriately direct and assist the staff under my control who will be involved in the study.

CHIEF INVESTIGATOR OR DELEGATE
SIGN ______________ DATE 28 / FEB / 2013
DR BRYAN MURRAY

The completed Protocol Agreement signifies review and acceptance of the protocol by the CI prior to initiation of the study and must be signed by both parties.

Once signed, the original must be kept on file by the Sponsor and the CI or delegate must retain a copy.

3 PROTOCOL SYNOPSIS

**PROTOCOL NUMBER**	RVL-CS-002
**SPONSOR**	Retroscreen Virology Ltd (RVL)
**STUDY TITLE**	A Randomised Single Blind Study to Characterise a Human Rhinovirus Type 16 for Use in Future Human Viral Challenge Studies
**PHASE**	Exploratory
**CHALLENGE VIRUS**	Human Rhinovirus (HRV-16 (RVL))
**ROUTE**	Intranasal drops
**SAMPLE SIZE**	Approximately 18 subjects will be studied in a quarantine environment of 3 groups (A, B, C) of approximately 6 subjects each with additional reserves.
**STUDY POPULATION**	Healthy male and/or female subjects aged 18 to 45 years inclusive who are sero-suitable for the Challenge Virus and meet the study eligibility criteria.
**INFECTIOUS TITRE**	HRV-16 (RVL) inoculum pre-diluted in a suitable diluent to give the appropriate nominal titre assigned randomised by Group pre inoculation:<br>Group A: 1 x $TCID_{50}$<br>Group B: 10 x $TCID_{50}$<br>Group C: 100 x $TCID_{50}$
**PRIMARY OBJECTIVE**	To determine a suitable infectious titre of HRV-16 (RVL) for use as a challenge virus in future Human Viral Challenge studies
**SAFETY OBJECTIVE**	The safety objective of the study is to assess the safety of the infectious HRV-16 (RVL) inoculum.
**SECONDARY OBJECTIVES**	The secondary objectives are:<br>• To discover biomarkers that can be used to develop a diagnostic platform to predict development of HRV-16 (RVL) illness in a population<br>• To characterise clinico-molecular and immunologic events during HRV-16 (RVL) infection<br>• To evaluate viral shedding from the nasal mucosa following challenge with HRV-16 (RVL)<br>• To evaluate symptoms following challenge with HRV-16 (RVL)<br>• To investigate the immunological response to infection with HRV-16 (RVL)

**PRIMARY ENDPOINT**	The primary endpoint of this study is the AUC of HRV-16 (RVL) virus shedding.
**SAFETY ENDPOINT**	The safety endpoint of the study is the incidence of virus challenge emergent adverse events (AEs) which are not consistent with a mild to moderate HRV-16 (RVL) infection.
**SECONDARY ENDPOINTS**	• Humoral and cell mediated immunity<br>• % of subjects infected with HRV-16 (RVL)<br>• AUC of symptoms<br>• Severity of symptoms<br>• % of subjects with fever<br>• % of subjects with:<br>○ URTI<br>○ LRTI<br>○ SRTI<br>○ Any illness<br>• % of subjects with Grade 2 or worse symptoms<br>• Clinical laboratory assessments<br>• Physical examination<br>• Spirometry<br>• ECG<br>• Immune response
**STUDY DESIGN**	**Screening:** Between 60 and 2 days (i.e. Day -60 to Day -3) prior to the day of inoculation with HRV-16 (RVL) volunteers will attend a screening visit during which their eligibility to participate will be assessed.<br><br>**Quarantine**: Eligible subjects will be admitted to the Quarantine Unit on Day -2 or -1 prior to the day of inoculation with HRV-16 (RVL) and will be assigned to one of three groups (A, B, C). Intranasal inoculation with HRV will be performed on Day 0 and subjects will remain in the Quarantine Unit until discharge on Day 8. If symptoms have not resolved on Day 8, subjects may be required to stay in isolation.<br><br>**Follow-up**: Subjects will attend for a Follow-up Visit on Day 28 (± 5d) post-inoculation. Each subject will be assessed by the CI or delegate for on-going symptoms and AEs.

STUDY ASSESSMENTS

- Medical history
- Height, Weight, Body Mass Index (BMI) and % body fat
- Urine: Class A drugs and cotinine urine screen
- Alcohol breath test
- Physical examination (complete and directed including ear and nose examinations)
- Vital signs: blood pressure (BP),respiratory rate (RR), and heart rate (HR), Oxygen saturation (SpO_2)
- Tympanic temperatures (^oC)
- 12 lead Electrocardiogram (ECG)
- Spirometry
- Tissue count and mucus weights
- RVL Symptom diary card
- HRV-16 (RVL) virus neutralisation assay
- Haematology, clinical biochemistry, coagulation, cardiac enzymes, and thyroid function test (TFT)
- Human Immunodeficiency Virus (HIV), Hepatitis B (HBV) and Hepatitis (HCV) screen
- Urinalysis
- Urine pregnancy test
- Beta Human Chorionic Gonadotrophin (β-HCG) blood pregnancy test (optional)
- Blood for biomarkers
- Throat swab (Tissue culture infective titre ($TCID_{50}$) and/or quantitative Polymerase Chain Reaction (qPCR)
- Nasal swabs ($TCID_{50}$ and/or qPCR)
- Nasal wash ($TCID_{50}$ and/or qPCR)
- AEs
- Prior and concomitant medication
- Nasal scrape (optional)
- Nasal brush (optional)
- Exhaled breath condensate may be collected for biomarkers and virus (optional)

VIROLOGY ASSAYS

Nasal wash, and/or nasal swabs, (nasal brushes and nasal scrape optional) samples will be analysed using HRV-16 (RVL) qPCR and/or HRV-16 (RVL) tissue culture ($TCID_{50}$), HRV-16 (RVL) virus neutralisation assay, and if required any other suitable virology assay, direct fluorescence antibody (DFA) assay for respiratory viral panel.

Blood will be processed for Peripheral Blood Mononuclear Cells (PBMC) and host Ribonucleic Acid (RNA).

STATISTICAL ANALYSIS

The safety population is defined as all subjects receiving HRV-16 challenge virus inoculum; this population will be used in presenting baseline and safety data. The safety analysis will be based on the safety population.

The virulence and pathogenicity population is defined as all sero-suitable subjects receiving HRV-16 challenge virus inoculum. The Per Protocol (PP) population is defined as all

virulence and pathogenicity population subjects who have no major protocol deviations and who complete the Quarantine period up to the final day of Quarantine, Day 8 (Discharge).

In terms of the primary analysis and other virulence and pathogenicity analyses, the primary concern will be the virulence and pathogenicity population, with analyses based on the PP population being of secondary concern. All virulence and pathogenicity analyses will be performed on both of these patient populations.

This is a randomised study. Subjects will be randomly allocated to groups A, B or C. Due to the size of the patient population in this study no statistical comparisons of groups are planned to be performed. The study will be summarised in terms of descriptive statistics.

The primary endpoint of this study is the AUC of HRV-16 (RVL) virus shedding, which will be summarised by group.

The safety endpoint of the study is the incidence of viral challenge emergent AEs which are not consistent with a mild to moderate HRV-16 (RVL) infection, which will be summarised by group. In addition, the incidence of all virus challenge emergent AEs will be reported within summary presentations, by system organ class and preferred term, and by group. Other safety parameters will be included within subject listings.

Descriptive statistics will be presented for each of the virulence and pathogenicity secondary endpoints by group.

4 ABBREVIATIONS AND DEFINITIONS

ABPI	Association of British Pharmaceutical Industries
ADR	Adverse Drug Reaction
AE	Adverse Event
ALP	Alkaline Phosphatase
ALT	Alanine Aminotransferase
AP	Analytical Plan
APTT	Activated Partial Thromboplastin Time
AST	Aspartate Aminotransferase
AUC	Area Under the Curve
βHCG	Human Chorionic Gonadotrophin
BD	Bis die (2 x daily)
BMI	Body Mass Index
BP	Blood Pressure
CI	Chief Investigator or delegate
COPD	Chronic Obstructive Pulmonary Disease
CK	Creatinine Kinase
CRO	Contract Research Organisation
CRP	C-Reactive Protein
CS	Clinically significant
CSR	Clinical Study Report
DBP	Diastolic Blood Pressure
DFA	Direct Fluorescence Antibody
DMP	Data Management Plan
DNA	Deoxyribonucleic Acid
ECG	Electrocardiogram
EIA	Enzyme immuno-assay
ELISA	Enzyme-Linked Immunosorbant Assay
FBC	Full Blood Count
FEF	Forced Expiratory Flow
FEV1	Forced Expiratory Volume
FI	Febrile Illness
FVC	Full Vital Capacity
GCP	Good Clinical Practice
GGT	Gamma Glutamyl Transferase
GMP	Good Manufacturing Practice
GP	General Practitioner
HBV	Hepatitis B Virus
HCV	Hepatitis C Virus
HIV	Human Immunodeficiency Virus
HR	Heart Rate
HRV-16 (RVL)	RVL manufactured Human Rhinovirus-16
ICH	International Conference on Harmonization
ICF	Informed Consent Form
IFN	Interferon
IMP	Investigational Medicinal Product
INR	International Normalised Ratio
ISF	CI or delegate’s Site File
IUD	Intra-Uterine Device
IUS	Intra-Uterine System
kg	Kilogram
LDH	Lactate Dehydrogenase
LRTI	Lower Respiratory Tract Infection
LSLV	Last Subject Last Visit

MCH	Mean Corpuscular Haemoglobin
MCHC	Mean Corpuscular Haemoglobin Concentration
MCV	Mean Corpuscular Volume
m	Metre
mL	Millilitre
mmHg	Millimetres of mercury
mRNA	Messenger RNA
NCS	Not clinically significant
OTC	Over-the-Counter
PBMC	Peripheral Blood Mononuclear Cell
PCR	Polymerase Chain Reaction
PP	Per Protocol
PT	Prothrombin Time
QA	Quality Assurance
qds	Quater in die (4 x per day)
qPCR	Quantitative Polymerase Chain Reaction
RBC	Red Blood Cell
REC	Research Ethics Committee
RNA	Ribonucleic Acid
RR	Respiratory Rate
RVAT	Rapid Virus Antigen Test
RVL	Retroscreen Virology Limited
SAE	Serious Adverse Event
SAP	Statistical Analysis Plan
SBP	Systolic Blood Pressure
SDV	Source Data Verification
SI	Systemic Illness
si-RNA	Short RNA
SNP	Single Nucleotide Polymorphisms
SOP	Standard Operating Procedure
SpO2	Oxygen saturation
SRTI	Systemic Respiratory Tract Illness
SSS	Study Specific Screening
$TCID_{50}$	Tissue Culture Infective Dose 50%(Titre)
tds	ter die sumendus (3 x daily)
TFT	Thyroid Function Test
URTI	Upper Respiratory Tract Infection
WBC	White Blood Cell
WHO	World Health Organisation

DEFINITIONS

Human Viral Challenge Study	A study to determine how a virus and the human body interact. Healthy subjects are isolated in the RVL Quarantine Unit and are infected (challenged) with a respiratory virus.
Non-IMP study	RVL subtype of study where no IMP is administered to subjects, although subjects are challenged with virus.
Titre	The titre of virus inoculum. The term 'titre' applies to virus inoculum or antibody.
Infectious titre	The titre of virus inoculum producing viral infection in a subject.
Quarantine Unit	RVL Isolation facility for Human Viral Challenge studies.
Quarantine Period	The period of time when clinical trial subjects are isolated in the Quarantine Unit during a Human Viral Challenge study.
Quarantine Group	A group of subjects resident in the Quarantine Unit who are receiving the same treatment regimen in a Human Viral Challenge study.
Seroconversion	Seroconversion is defined as a more than or equal to 4 fold increase in antibodies to HRV-16 from baseline.
Symptom Diary	Document in which the subject records their assessment of symptoms related to the study indication.
Virulence and pathogenicity population	All sero-suitable subjects receiving virus inoculum.
Per Protocol (PP) Population	For a non-IMP study, the PP population is defined as all virulence and pathogenicity population subjects who have no major protocol deviations and who complete the Quarantine period up to the final day of Quarantine.
Safety Population	For a non-IMP study, the safety population is defined as all subjects receiving challenge virus inoculum. The sero-suitability of the subject for the study, which may only be determined after viral challenge, has no bearing on inclusion of the subject in the safety population.
Viral challenge (or challenge)	The inoculation of a study subject with virus inoculum. By definition, the day of viral challenge is Day 0.
End of study	The end of the study is defined as last subject last visit (LSLV)
Chief CI or delegate	The CI or delegate responsible for conducting the trial (GCP E6(R1) 1996 Section 6.1.5)

Sponsor's Medical Expert	An appropriately qualified medical person designated by the Sponsor who will be readily available to advise on trial related medical questions or problems.
Monitor/Monitoring Organisation	The company overseeing progress of the clinical trial and ensuring that it is conducted, recorded, and reported in accordance with the protocol, standard operating procedures (SOPs), GCP and applicable regulatory requirements (GCP E6 (R1) 1996 Section 1.38).<br><br>This monitoring function may be fulfilled by the Sponsor or be contracted out to a Contract Research Organisation (CRO).
Study Monitor	The individual assigned by the Monitoring Organisation to perform study monitoring visits to the (investigational) study site.

5 INTRODUCTION

Retroscreen Virology Ltd (RVL) is an independent specialist research company with its origins in the University of London. RVL primarily conducts studies into antiviral chemotherapy and vaccine development. Potential studies can range from single vaccine immunogenicity to field studies (where people with respiratory virus infections are identified). In addition, the company conducts Human Viral Challenge studies in which subjects are given a vaccine (or placebo) then inoculated (challenged) with a respiratory virus in a controlled quarantine facility.

RVL has conducted over 46 quarantine studies and safely inoculated over 959 volunteers. The Company's research has demonstrated proof of concept for novel therapeutics such as DNA vaccines [1], siRNAs[2], and also early stage diagnostic platforms[3,4].

All studies are individually submitted for favourable ethics committee opinion and, if required, regulatory approval.

5.1 The Challenge Virus: Human Rhinovirus (HRV)

Rhinoviruses are one of the causative agents for upper respiratory tract infection and are important predisposing factors to associated conditions such as sinusitis, otitis media, bronchitis and primary pneumonia[5]. Rhinovirus infections may trigger asthma attacks in children and the association may persist in many adults with asthma. At present, rhinovirus is the number one cause of asthma exacerbations [6].

Rhinovirus infection may also precipitate serious infection in the elderly and there is strong epidemiological evidence of a relationship between rhinovirus infection and exacerbation of asthma and COPD[7]. Epidemiological studies have demonstrated that a significant number of pulmonary exacerbations of Cystic Fibrosis are also preceded by rhinovirus infections. Each episode of infection appears to cause the progression of the underlying disease and as each exacerbation actually shortens the life of the patient, infection is potentially life-threatening [8-10].

To date, no antiviral agent has been approved for the treatment of rhinovirus infection, and clinical treatment is directed towards addressing symptoms of the disease. There is therefore a need for the development of effective therapies for rhinovirus infection, which could reduce the serious health effects in the vulnerable populations described above. Furthermore, effective treatment of rhinovirus infection in the healthy population could also result in an improvement in sickness records, with a consequent increase in productivity over the winter months.

A wide variation of infectious titres of HRV has been used in earlier studies and the clinical syndrome associated with experimental infection is well-described. Similar viruses have also been used to induce experimental rhinovirus infections in approximately 600 adult volunteers [11,12].

Challenge with HRV generally produces infection in up to 90% of susceptible volunteers. Symptoms usually first appear within 24 hours after inoculation and peak at 48-72 hours after challenge. The clinical syndrome is comparable to that reported in natural colds [11] although

in one study with identical definitions for duration the median duration of illness was 3.5 days in volunteers with experimental colds and 5.5 days in volunteers with natural colds. Approximately one-third of rhinovirus infections, whether natural or experimental, are asymptomatic. Virus shedding in infected volunteers follows a pattern similar to that of the symptoms. Virus concentrations in nasal lavage fluid generally peak 2-3 days after challenge and then rapidly decrease.

5.2 Rationale for Study

The purpose of this study is to identify the optimal infectious titre of HRV-16 (RVL) for use in future clinical studies. A suitable infectious titre would be one that yields infection in approximately 60 to 90 % of volunteers along with mild to moderate clinical symptoms in the majority of those infected.

Previously, challenge of volunteers with HRV-16 (non-RVL studies) generally produced infection in up to 90% of susceptible volunteers. Symptoms usually first appear within 24 hours after inoculation and peak at 48-72 hours after challenge. Approximately one-third of HRV infections, whether natural or experimental, are asymptomatic. Virus shedding in infected volunteers follows a pattern similar to that of the symptoms. Virus concentrations in nasal lavage fluid generally peak 2-3 days after challenge and then rapidly decrease.

5.3 Potential Risks

Healthy adult male and/or female subjects will be enrolled as they are best suited to tolerate inoculation with the challenge virus.

Subjects have approximately 60% to 90% chance of becoming infected with mild symptoms of HRV-16 (RVL) following inoculation. Typical HRV illness is characterised by a slow onset of rhinitis, nasal stuffiness, fever; malaise, myalgia (muscle aches), and sore throat. In healthy adults the illness usually resolves without any treatment, with relief of symptoms occurring naturally within 7 days.

As complications occur in those with pre-existing conditions such as asthma, individuals with asthma will be excluded from the study.

It is unlikely that subjects will transmit HRV-16 (RVL) to their close contacts as the usual duration of HRV infection is less than the time subjects will spend in Quarantine. Therefore, although the virus will be present in subjects' noses for several days after infection with HRV, once they leave the isolation facility there should not be sufficient HRV-16 (RVL) in their noses to transmit to others.

However, as a further safety measure subjects will be instructed to avoid contact with the following groups of people for two weeks after they leave the isolation facility:

- has known immunodeficiency
- is receiving immunosuppressant medication
- is undergoing or soon to undergo cancer chemotherapy within 28 days of viral inoculation

- has been diagnosed with emphysema or chronic obstructive pulmonary disease (COPD), is elderly and resides in a nursing home, or who has severe lung disease or a medical condition that may include but not exclusive to the conditions listed in Appendix 2
- has received a bone marrow or solid organ transplant

There are no anticipated benefits to the subjects in this study.

6 STUDY OBJECTIVES AND ENDPOINTS

6.1 Primary Objective

To determine a suitable infectious titre of HRV-16 (RVL) for use as a challenge virus in future Human Viral Challenge studies.

6.1.1 Primary Endpoint

The primary endpoint of the study is AUC of HRV-16 (RVL) virus shedding.

6.2 Safety Objective

To assess the safety of the infectious HRV-16 (RVL) inoculum.

6.2.1 Safety Endpoint

The safety endpoint of the study is the incidence of virus challenge emergent AEs which are not consistent with a mild to moderate HRV-16 (RVL) infection.

6.3 Secondary Objective(s)

The secondary objectives are:

- To discover biomarkers that can be used to develop a diagnostic platform to predict development of HRV-16 (RVL) illness in a population
- To characterise clinico-molecular and immunologic events during HRV-16 (RVL) infection
- To evaluate viral shedding from the nasal mucosa following challenge with HRV-16 (RVL)
- To evaluate symptoms following challenge with HRV-16 (RVL)
- To investigate the immunological response to infection with HRV-16 (RVL)

6.3.1 Secondary Endpoints

- Humoral and cell mediated immunity
- % of subjects infected with HRV-16 (RVL)
- AUC of symptoms
- Severity of symptoms
- % of subjects with fever
- % of subjects with:
  - URTI
  - LRTI
  - SRTI
  - Any illness
- % of subjects with Grade 2 or worse symptoms
- Clinical laboratory assessments
- Physical examination
- Spirometry
- ECG

- Immune responses

7 STUDY POPULATION

Potential subjects will be identified from RVL's volunteer database.

A blood sample for HRV-16 (RVL) antibody determination will be taken for all potential subjects at the study-specific screening visit (Day -60 to Day -3). One repeat pre-challenge (Day -2 and Day -1) sample will be drawn to confirm sero-suitability. Considering the elapsed time required for results availability, it is acceptable to exclude sero-unsuitable volunteers in post-hoc fashion, from the virulence and pathogenicity analysis.

Approximately 18 subjects will be studied in three groups (each with approximately 6 subjects) in a quarantine environment. Eligible subjects will be healthy males and/or females aged 18 to 45 years inclusive, who are sero-suitable for HRV-16 (RVL) and meet the eligibility criteria described in Sections 7.1 and 7.2.

7.1 Inclusion Criteria

MAIN INCLUSION CRITERIA				
**Inclusion can be assessed and finalised on the following study time points marked by an 'X'**		**SSS**	**Day -2/-1**	**Day 0**
1	Age 18 to 45 years.	**X**		
2	An informed consent document signed and dated by the subject and CI or delegate, confirming their comprehension and their ability to comply with the study requirements.	**X**		
3	Healthy males and females, (healthy defined as no clinically significant abnormalities identified by a detailed medical history, full physical examination, vital signs, 12 lead ECG, and clinical laboratory tests) at screening by the CI or delegate.	**X**		
4	Body mass index (BMI [kg/m^2] = Body weight [kg] ÷ Height^2 [m^2]) of 18 to 33 kg/m^2; and a total body weight ≥50 kg.<br>If BMI is >30 kg/m^2, a body fat percentage will be performed.	**X**		
5	Sero-suitable for challenge virus.	**X**	**X**	**X**
6	Subjects must agree to use acceptable contraceptive methods if they are heterosexually active (Appendix 1)<br>• Females of child bearing potential must agree to use a protocol-recommended method of contraception from the screening visit throughout the study period until the last visit.<br>Sterilised male and/or female subjects will be required to provide evidence of this.	**X**	**X**	

7.2 Exclusion Criteria

MAIN EXCLUSION CRITERIA				
**Exclusion can be assessed and finalised on the following study time points marked by an 'X'**		**SSS**	**Day -2/-1**	**Day 0**
**1**	Health care workers (including doctors, nurses, medical students and allied healthcare professionals) anticipated to have patient contact within two weeks of human viral challenge. Healthcare workers should not work with patients until 14 days after challenge or until their symptoms are fully resolved (whichever is the longer). In particular, any health care workers who work in units housing elderly, disabled or severely immuno-compromised patients (e.g. bone marrow transplant units) will be excluded from working or participating in the study.	**X**		
**2**	Those employed or immediate relatives of those employed at Retroscreen Virology.	**X**		
**3**	Presence of household member or close contact (for an additional 2 weeks after discharge from the isolation facility) who is:<br>• any person with any known immunodeficiency;<br>• any person receiving immunosuppressant medications;<br>• any person undergoing or soon to undergo cancer chemotherapy within 28 days of viral inoculation;<br>• Any person who has diagnosed emphysema or chronic lung condition, is elderly residing in a nursing home, or with severe lung disease or medical condition that may include but not exclusive to the conditions listed in Appendix 2;<br>• Any person who has received a transplant (bone marrow or solid organ).	**X**		
**4**	Known allergy to components of the challenge virus inoculum.	**X**		
**5**	Presence of any significant acute or chronic medical illness as listed in Appendix 2– Excluded Medical or Psychiatric conditions, that in the view of the CI or delegate(s), is associated with increased risk of complications of respiratory viral illness or make the subject unsuitable for a Quarantine challenge study.<br><br>To include but not exclusive of: Any significant abnormality altering the anatomy of the nose or nasopharynx observed by physical examination.	**X**		

MAIN EXCLUSION CRITERIA				
**Exclusion can be assessed and finalised on the following study time points marked by an 'X'**		**SSS**	**Day -2/-1**	**Day 0**
**6**	Loss (including blood donations) of 450 mL or more of blood, during the 3 months prior to inoculation.		**X**	
**7**	Receipt of any investigational drug within 3 months prior to inoculation, or any other respiratory human viral challenge within 1 year prior to inoculation, or receipt of more than 4 investigational drugs within the previous 12 months.		**X**	
**8**	• Subjects who have a significant history of any tobacco use at any time (≥ total 10 pack year history, e.g. 10.1 etc. would be excluded) and<br>• Are unwilling to desist from smoking for a period up to quarantine admission as evidenced by a negative urinary cotinine test.	**X**	**X**	
**9**	Receipt of prior or Concurrent Medications as detailed (but not limited to) in Section10.1, that in the view of the CI or delegate(s), makes the subject unsuitable for a challenge study.	**x**	**x**	
**10**	Any other finding in the medical interview, physical exam, screening investigations or from the General Practitioner (GP) that, in the opinion of the CI or delegate deems the subject unsuitable for the study.	**x**	**x**	
**11**	Serology - Positive Human Immunodeficiency Virus (HIV), Hepatitis B (HBV) or Hepatitis C (HCV) assay.	**x**	**x**	
**12**	Symptomatic subjects:<br>• Subjects symptomatic with hay fever between admission and inoculation.<br>• Presence of any significant respiratory symptoms existing on the day of inoculation or between admission for Quarantine and viral inoculation.<br>• History suggestive of respiratory infection within 14 days prior to admission into the Quarantine Unit.		**x**	
**13**	Subjects who are pregnant or nursing, or have a positive pregnancy test.	**x**	**x**	**x**
**14**	A history or evidence of any of the below:<br>• drugs of abuse<br>• positive urine Class A drug test or<br>• alcohol breath screen<br>-that cannot be satisfactorily explained (e.g. recent mouthwash, use of codeine / poppy seeds)	**x**	**x**	**x**

7.3 Subject Numbering

Potential subjects will be assigned a RVL subject number which will be used to identify subjects up to the point of viral challenge. A blinding baseline number will be assigned just before the viral challenge.

7.4 Subject Withdrawal Criteria

A subject can withdraw from the study at any time, for any reason, without prejudice to their future medical care. Every reasonable effort will be made to ascertain the reason for withdrawal, and if known will be recorded on the source documents. If appropriate, the subject will be encouraged to remain available for follow-up medical monitoring, and a complete Day 28 (± 5d) Follow-up Visit assessment should be completed. The Sponsor's representative will be notified as soon as possible.

Subjects who withdraw from the study prematurely after viral challenge are required to ensure that susceptible contacts are not exposed to residual virus. Susceptible contacts include a household member or close contact (for an additional two weeks after the discharge from the isolation facility) that is:

- Less than 3 years of age
- Any person with any known immunodeficiency
- Any person receiving immunosuppressant medications
- Any person undergoing or soon to undergo cancer chemotherapy within 28 days of challenge
- Any person who has diagnosed emphysema, asthma or COPD, is elderly residing in a nursing home, or has severe lung disease
- Is elderly and residing in a nursing home
- Any person who has received a transplant (bone marrow or solid organ).

Subjects who are withdrawn or discontinue after challenge will not be replaced.

7.5 Discontinuation of Subject Participation

Participation by individual subjects in this study may be discontinued for any of the following reasons:

- Subject request to withdraw from further participation
- Intolerable AEs
- The subject needs to take medication(s) which may interfere with the study assessments
- Subject non-compliance with the protocol requirements
- Continuation in the study would be detrimental to the subject's safety in the opinion of the CI or delegate
- Clinically significant abnormal laboratory findings (which in the opinion of the CI or delegate precludes further participation in the study)
- Development of intercurrent illness which, in the opinion of the CI or delegate, would compromise the health of the subject or the study objectives

Every attempt will be made to encourage subjects who discontinue after virus challenge to comply with study procedures until the Day 28 (± 5 days) Follow-up Visit, in order to ensure their safety and allow for optimal inclusion of study data in the database. Subjects who discontinue after undergoing Human Viral Challenge will not be replaced.

In the event that a subject discontinues prematurely due to an AE or SAE, the subject will be followed up until the AE or SAE resolves or stabilises, or is judged by the CI or delegate to be no longer clinically significant. If necessary, the subject may be referred to their GP or a specialist, as appropriate.

7.6 Discontinuation of the Study

RVL as the Sponsor reserves the right to end the study at any time. In case of premature termination or suspension of the study, RVL will promptly inform the CI and (if applicable), regulatory authorities of the termination or suspension, providing the reason(s). It is the responsibility of the CI, however, to notify the REC in case of premature termination or suspension of the study.

The CI or Sponsor may terminate this study for reasonable cause before the expiration of the agreed time period, provided a written notice to the REC is submitted at a reasonable time in advance of intended termination.

8 STUDY DESIGN

8.1 Summary of Study Design

This is a Phase I, randomised single blind study to identify a suitable infectious titre of HRV-16 (RVL) for use in future Human Viral Challenge studies. Subjects who meet the eligibility criteria described in Section 7 will be enrolled.

Figure 1 depicts the study design for participating subjects. There are three sequential phases to the study:

- Screening
- Quarantine and challenge phase
- Follow-up

The days and times of assessments during the study are detailed in Table 2: Time and Events Schedule.

Figure 1: Study Design for Subjects

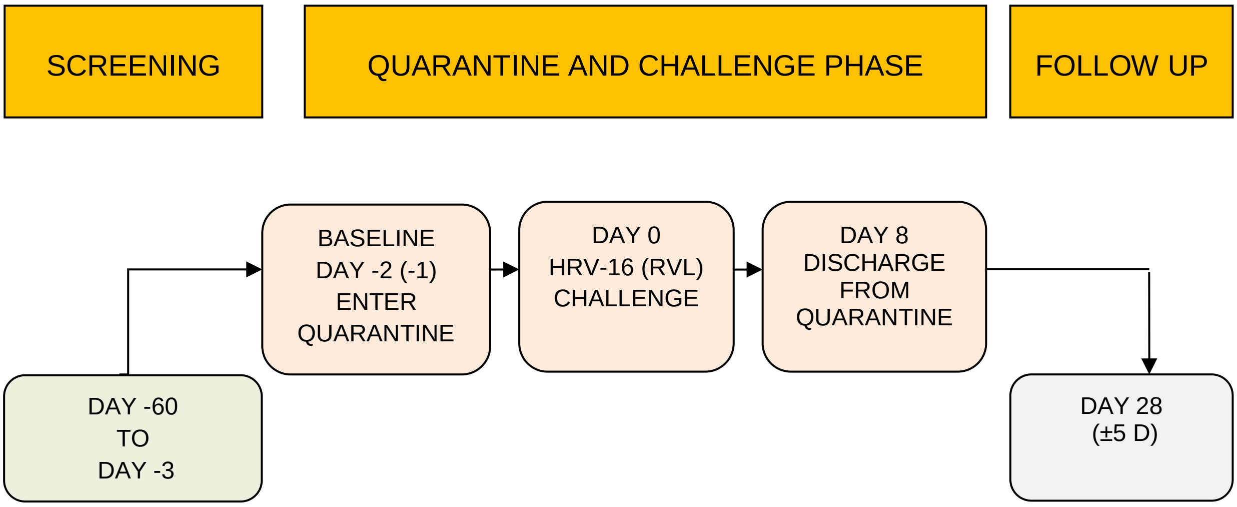

8.1.1 Screening

Between Day -60 and -3 (prior to viral challenge), approximately 131 eligible subjects will be invited to SSS and approximately 41 are likely to be eligible for the quarantine phase.

A screening log will be completed for all subjects who sign an ICF at SSS and a subject identification log will be completed for all subjects who are randomised.

Subjects who do not meet the study criteria at Screening may be re-screened, in whole or in part depending on the reason for exclusion.

8.1.2 Quarantine and Challenge Phase

Eligible subjects will enter the Quarantine Unit on Day -2 or Day -1 and will be observed for symptoms of illness. Any subject who develops symptoms of illness may be asked to leave the Quarantine Unit and be replaced by a reserve subject.

Randomised subjects will be studied within a quarantine environment allocated to one of three groups. Each group will receive one of the three selected titres of HRV as shown in Table 1: Challenge Virus Titre for Quarantine Groups

Subjects will receive the intranasal inoculation of HRV-16 (RVL) on Day 0 and will remain in Quarantine for 8 days. Subjects in a quarantine group may not all be inoculated on the same day.

Table 1: Challenge Virus Titre for Quarantine Groups

Quarantine Group Number	Virus Titre	Number of volunteers
1	1 x $TCID_{50}$	6
2	10 x $TCID_{50}$	6
3	100 x $TCID_{50}$	6

During the Quarantine period, assessments and procedures will be performed as described in Table 2: Time and Events Schedule. Subjects who have symptoms or on-going AEs on the day of discharge (Day 8) may be required to stay an additional night at the CI's discretion for additional safety monitoring until resolution of symptoms.

8.1.3 Follow-up Visit

Subjects will return for a final end of study Follow-up Visit on Day 28 (± 5 days) post-HRV-16 (RVL) inoculation.

8.2 Duration of the Study

The end of the study is defined as last subject last visit (LSLV). The duration of the entire clinical trial is expected to be no more than 94 days from screening to LSLV, (unless subjects have to stay in isolation for additional days or require extended follow up for unresolved AEs).

Any AEs that are unresolved at the Day 28 post-challenge Follow-up Visit may necessitate further follow-up visits up to 60 days post-challenge, in which case the maximum duration of the clinical trial for a subject could be up to 121 days (Day -60 to Day 60).

The duration of the entire clinical trial is expected to be approximately one year.

9 CHALLENGE VIRUS

9.1 Production

The challenge virus HRV-16 (RVL) was produced by cell culture passage of a rhinovirus isolated from a human volunteer who was challenged with another HRV-16 virus stock. The nasal wash specimen collected for virus isolation was frozen in cryovials and stored at -80°C. One vial formed the starting material for the development of the challenge virus pool.

HRV-16 (RVL) is manufactured in compliance with the standards of GMP and tested in accordance with standards for licensed live viral vaccines. The documentation associated with the virus production was reviewed by RVL's Quality Assurance (QA) Department.

All preparation of HRV-16 (RVL) for the study will be performed according to the RVL Analytical Plan (AP) as per RVLs standard procedures. The final product will be supplied as a separate aliquot for each volunteer.

9.2 Storage and Accountability

Long term storage of the challenge virus should be at <= -70°C in a secured and alarm-monitored freezer.

The CI or delegate will maintain accurate records of the receipt, condition and dispensing details of all HRV-16 (RVL) inoculum stock. Any departure from the dispensing regimen will be recorded.

HRV-16 (RVL) inoculum accountability records will be available for verification by the Study Monitor.

9.3 Administration

The HRV-16 (RVL) inoculum shall be prepared and administered in accordance with RVL's SOPs. Each subject will receive intranasal administration of HRV-16 (RVL) inoculum pre-diluted in a suitable diluent to give the appropriate nominal titre for each Quarantine group. The inoculum will be delivered in drops.

9.4 Unused Challenge Virus

All unused challenge virus must be retained until verification and accountability procedures have been performed. Challenge virus that is no longer required and materials that come into contact with challenge virus may be stored in the RVL Biorepository.

10 STUDY RESTRICTIONS

10.1 Concomitant Medications

Additional concomitant medications not listed below will be assessed by the CI or delegate and exclusion will only be made on the basis that the concomitant medication would affect the volunteer's suitability to receive the Challenge Virus inoculum.

Excluded prior or current medications are as follows:

- Any medication for symptoms of hay fever, rhinitis, nasal congestion or respiratory tract infection within 7 days prior to human challenge inoculation
- Any other product (prescription or over-the-counter), for symptoms of hay fever, rhinitis, nasal congestion or respiratory tract infection within 7 days prior to viral challenge
- Systemic glucocorticoids within 1 month
- Antiviral drugs within 1 month
- Immunoglobulin's (Igs) within 1 month
- Receipt of blood transfusions/ blood products within 1 month
- Receipt of any other cytotoxic- within 6 months prior to dosing
- Receipt of any other immunosuppressive drug within 6 months prior to prior to viral challenge
- Receipt of any systemic chemotherapy agent at any time

Any medications taken during the 30 days prior to viral challenge will be recorded in the subject's source documents.

Thereafter and during the study periods the CI or delegate may permit a limited amount of Paracetamol (no more than 4 g per day) or topical medication (maximum amount to be judged by the CI) for the treatment of headache or any other pain. Other medication to treat AEs may be prescribed if required.

Any concomitant medication required for the subject's welfare may be given by the CI or his designee. The CI or designee will ensure that details regarding the medication and their reason for use are recorded in the source documentation.

Any changes in medications during the study will also be recorded.

10.2 Other Restrictions

The following are forbidden from 72 hours (3 days) before entry to Quarantine and during Quarantine:

- alcohol
- smoking
- use of other tobacco products

Subjects will also be advised to avoid strenuous activities from Screening until the last study visit.

11 STUDY PROCEDURES

All procedures will be undertaken in compliance with the standards and principles of GCP.

11.1 Time and Events Schedule

Details of the timing of Assessments and Procedures can be found in Table 2: Time and Events Schedule below. As per the Time and Events schedule those procedures marked as optional can be performed at the CI’s discretion.

Table 2: Time and Events Schedule

Study Phase>	Screening	Check In		QUARANTINE ISOLATION										Discharge	Follow Up	Withdrawal Visit
Study Day >	Day -60 to Day -3	Day -2	Day -1	Day 0 : Viral Inoculation			Day	Day	Day	Day	Day	Day	Day	Day	Day	Days
Procedure		Admission to quarantine		Day 0: Pre challenge	Day 0: Challenge	Day 0: Post challenge	1	2	3	4	5	6	7	8	28 (± 5d)	0-7
Informed consent	X															
Demographics	X															
Medical history and prior medications	X	X[a]	X[a]													
Eligibility criteria	X	X[a]	X[a]	X												
Randomisation				X												
Adverse Events and Concomitant Medications	X	X[a]	X	X	X	X	X	X	X	X	X	X	X	X	X	(X)
Blood Cardiac enzymes	X	X[a]	X[a]	(X)			(X)	(X)	X	(X)	(X)	(X)	X	(X)	X	(X)
Blood Coagulation	X	X[a]	X[a]	(X)			(X)	(X)	X	(X)	(X)	(X)	X	(X)	X	(X)
Blood Glucose level	X	X[a]	X[a]	(X)			(X)	(X)	X	(X)	(X)	(X)	X	(X)	X	(X)
Blood Haematology and Chemistry	X	X[a]	X[a]	(X)			(X)	(X)	X	(X)	(X)	(X)	X	(X)	X	(X)
Blood HIV, Hepatitis B & C serology	X															
Blood HRV-16 (RVL) neutralisation assay	X	X[a]	X[a]												X	
Blood: PBMC and PAXgene		X[a]	X[a]						X				X		X	(X)
Blood Pregnancy test β-HCG		(X)														
Blood Thyroid Function Test	X	X[a]	X[a]													
Body Fat/BMI Height[b]	X														X	(X)

Notes: **(X)** At the CI or delegate's discretion; **TDS**- Three times daily (approximately 8 hourly); **BD** -Twice daily; **a**-Procedures may be conducted on either Day -2 or Day -1; **b**-Height at screening only; **c**-At the CI or delegate's discretion, this could be a directed physical examination, including otoscopy. Subjects will be reviewed by a Physician regarding HRV symptoms or URTI; **d**- Tympanic temperature will be recorded 3 x daily, from midday on Day -2 until discharge from Quarantine (± 2 measurements); **e**-For tolerance only; **f**-Optional; **g**- No $TCID_{50}$ or qPCR to be performed.

Study Phase>	Screening	Check In		QUARANTINE ISOLATION										Discharge	Follow Up	Withdrawal visit
Study Day >	Day -60 to Day -3	Day -2	Day -1	Day 0 : Viral Inoculation			Day	Day	Day	Day	Day	Day	Day	Day	Day	Days
Procedure		Admission to quarantine		Day 0: Pre challenge	Day 0: Challenge	Day 0: Post challenge	1	2	3	4	5	6	7	8	28 (± 5d)	0-7
Breath Alcohol test	X	X[a]	X[a]	(X)			(X)	(X)	(X)	(X)	(X)	(X)	(X)	X	(X)	(X)
Breath Exhaled condensate[f]				(X)		(X)	(X)	(X)	(X)	(X)	(X)	(X)	(X)	(X)		(X)
ECG 12 Lead	X	X[a]	X[a]										X		(X)	
Nasal Brush and/or Scrape ($TCID_{50}$ and/or qPCR)[f]	(X)[e]	(X)												(X)		
Nasal Swab: Respiratory virus screen (DFA)		X[a]	X[a]													
Nasal Swab ($TCID_{50}$ and/or qPCR)	X[e]						TDS							X	(X)	(X)
Nasal Wash ($TCID_{50}$ and/or qPCR)	X[e]	X[a]	X[a]				BD							X	(X)	(X)
Complete Physical Examination[c]	X	X[a]	X[a]											X	X	(X)
Directed Physical Examination				X		X	X	X	X	X	X	X	X			
Spirometry	X	X[a]	X[a]	X			(X)	(X)	(X)	(X)	(X)	(X)	(X)	(X)	(X)	(X)
Symptom Diary Card		X	BD	X		X	TDS							X		(X)
Throat Swab ($TCID_{50}$ and/or qPCR)	X[e]	X[g]							X				X	X		(X)
Tissue Count and mucus weight				X			X	X	X	X	X	X	X	X		(X)
Tympanic Temperature[d]	X	X[a]	X[a]	TDS										X	X	(X)
Urinalysis (dipstick)	X	X[a]	X[a]						X				X		X	(X)
Urine Class A Drug and cotinine screen	X	X[a]	X[a]	(X)			(X)	(X)	(X)	(X)	(X)	(X)	(X)			(X)
Urine Pregnancy test	X	X[a]	X[a]	X										X	X	(X)
Virus Challenge Inoculation HRV-16 (RVL)					X											
Vital signs (BP, RR, HR and SpO_2)	X	X	TDS											X	X	(X)

Notes: **(X)** At the CI or delegate's discretion; **TDS**- Three times daily (approximately 8 hourly); **BD** -Twice daily; **a**-Procedures may be conducted on either Day -2 or Day -1; **b**-Height at screening only; **c**-At the CI or delegate's discretion, this could be a directed physical examination, including otoscopy. Subjects will be reviewed by a Physician regarding HRV symptoms or URTI; **d**- Tympanic temperature will be recorded 3 x daily, from midday on Day -2 until discharge from Quarantine (± 2 measurements); **e**-For tolerance only; **f**-Optional; **g**- No $TCID_{50}$ or qPCR to be performed.

11.2 Screening Visit (Day -60 to Day -3)

The Screening Visit (Day –60 to -3) will occur in a clinic environment. The subjects will be invited if they are confirmed sero-suitable (likely to become infected with the HRV-16 (RVL) virus). The following procedures will be performed.

- Written informed consent (Section 19.9.2.)
- Demographics
- Eligibility criteria
- Medical history
- Prior medications

The subject may be excluded prior to the below procedures being performed if the investigators find the subjects ineligible during their consenting consultation. The subject will then proceed to have the following procedures performed, but may be excluded at any point, should a procedure immediately demonstrate ineligibility.

- AEs and Concomitant Medications
- Blood samples
- Blood glucose level
- Haematology and biochemistry; coagulation; cardiac enzymes, TFT
- HRV-16 (RVL) viral neutralisation assay
- HIV, HBV and HCV screening
- Breath alcohol test
- Body fat, height and weight, BMI
- 12 Lead ECG
- Nasal brush (Optional)
- Nasal scrape (Optional)
- Nasal swab (Tolerance only)
- Nasal wash (Tolerance only)
- Spirometry
- Throat swab (Tolerance)
- Complete physical examination
- Tympanic temperature
- Urinalysis
- Urine Class A drugs and cotinine
- Urine Pregnancy (females only)
- Vital signs: Blood pressure (BP), respiratory rate (RR), heart rate (HR), pulse oximetry (SpO_2)

11.3 Day -2 and Day -1

The following procedures will be performed for each subject as indicated below on either Day -2 or Day -1 in a Quarantine Environment.

- Medical history and prior medications – Day -2 or Day -1
- Eligibility criteria Day - 2 or Day -1
- AEs and Concomitant medications – Day -2 and Day -1
- Blood – β HCG – Day -2 or Day -1
- Blood – Biochemistry including Cardiac Enzymes, and TFT - Day -2 or Day -1
- Blood – Haematology and Coagulation Day -2 or Day -1
- Blood – Glucose level- Day -2 or Day -1
- Blood – HRV-16(RVL) Viral Neutralisation Assay Day -2 or Day -1
- Blood – PBMC and RNA – Day -2 or Day -1
- Breath alcohol test - Day -2 or Day -1
- 12 Lead ECG - Day -2 or Day -1
- Complete physical examination - Day -2 or Day-1
- Nasal scrape – Optional (not to be performed on Day -1)
- Nasal brush – Optional (not to be performed on Day -1)
- Nasal swab – Respiratory virus screen (DFA) - Day -2 or Day -1
- Nasal wash – ($TCID_{50}$ and/or qPCR) - Day -2 or Day -1
- Spirometry – Day -2 or Day -1
- RVL Symptom diary card – Once daily on Day -2, twice on Day -1
- Throat swab – Day -2
- Tympanic temperature – Day -2 and Day -1 up to three times a day
- Urinalysis – Day -2 or Day -1
- Urine – Class A Drugs (Generic Kit) and Cotinine – Day -2 or Day -1
- Urine – Pregnancy test (females only) – Day -2 or Day -1
- Vital signs: BP, RR, HR and SpO_2 - Day -2 and up to three times a day on Day -1

The data will be reviewed with respect to the eligibility criteria:

- Eligible subjects will remain resident in the Quarantine Unit.
- Subjects who no longer fulfil all of the enrolment criteria, have evidence of a respiratory infection or are deemed by the CI or delegate to be unfit to be inoculated with the challenge virus will be discharged and replaced with reserve subjects, Subjects who are withdrawn will be asked to complete the last visit and safety follow-up as required.
- SSS re-screening will not be required for reserve subjects, however they will need to attend the full quarantine from Day -2/ Day -1. If they fall outside the screening period a full re-screening will be required.

11.4 Viral Challenge (Day 0)

11.4.1 Pre-challenge

Once subjects are confirmed fully eligible on the morning of inoculation, they will be assigned a randomisation code by the RVL laboratory. The CI and clinical team will remain blinded to the randomised inoculum titre to be given to each subject per group.

- AEs and concomitant medications
- Eligibility
- Directed physical examination
- Randomisation
- Spirometry
- RVL Symptom diary card
- Tympanic temperature
- Tissue count and mucus weight (initial supply given on Day -1 AM)
- Urine- Pregnancy test (females)
- Vital signs – BP, RR, HR, SpO_2

- Breath exhaled condensate (optional)

During the Quarantine period, additional safety tests (e.g. Urine Class A drugs and cotinine, breath alcohol, spirometry, ECG, blood safety tests) may be undertaken at the Study Physician's discretion.

11.5 Day 0

11.5.1 Viral Challenge

- Inoculation - Administration of intranasal HRV-16 (RVL)
- AEs and concomitant medications

11.5.2 Post-challenge

The following will be conducted once on the evening after intranasal inoculation:

- Adverse events and concomitant medication
- Directed physical examination
- RVL Symptom diary card
- Tympanic temperature
- Vital signs: BP, RR, HR and SpO_2
- Breath exhaled condensate (optional)

11.6 Quarantine Study Days 1 to 7

The following procedures and assessments will be performed on Days 1 to 7 inclusive, as per the Table 2: Time and Events Schedule.

The following procedures and assessments will be performed on Days 1 to 7 inclusive unless otherwise indicated below.

- Adverse events and concomitant medications
- Directed physical examination (AM)
- Nasal swab ($TCID_{50}$ and/or qPCR) - three times a day
- Nasal wash ($TCID_{50}$ and/or qPCR) - twice a day
- Spirometry – optional
- RVL Symptom diary card - three times a day
- Tissue count and mucus weight
- Tympanic temperature – three times a day
- Vital signs: BP, RR, HR and SpO_2 - three times a day
- Breath exhaled condensate (optional)

Assessments that are scheduled as two or three times daily (symptom diary card, nasal swab/ or wash, vital signs and temperature) will be carried out at approximately the same time points.

11.6.1 Exceptions

Other assessments will also be performed as per Table 2: Time and Events Schedule.

- Blood - Biochemistry including Cardiac Enzymes - Days 3 and 7 only
- Blood - Coagulation - Days 3 and 7 only
- Blood - Glucose level - Days 3 and 7 only
- Blood - Haematology - Days 3 and 7 only
- Blood - PBMC and RNA - Days 3 and 7 only
- ECG - 12 lead - Day 7 only
- Throat swab ($TCID_{50}$ and/or qPCR) Days 3 and 7only
- Urinalysis - Days 3 and 7 only

11.7 Discharge from Quarantine (Day 8)

Subjects who are deemed well at on the morning of Day 8 may be discharged from the Quarantine Unit, at the discretion of the CI or delegate. If the subject is not symptom free, a further night(s) may be required (at the CI’s discretion) to confirm if the subject is suitable for discharge into the community.

The following procedures will be performed on Day 8 prior to discharge:

- Adverse events and concomitant medications
- Breath alcohol test
- Nasal swab ($TCID_{50}$ and/or qPCR)
- Nasal wash ($TCID_{50}$ and/or qPCR)
- Complete physical examination

- Spirometry - optional
- RVL Symptom diary card
- Throat swab ($TCID_{50}$ and/or qPCR)
- Tissue count and mucus weight
- Tympanic temperature
- Urine pregnancy test (females)
- Vital signs: BP, RR, HR and SpO_2
- Breath exhaled condensate (optional)

Subjects will be informed how to contact the CI or delegate (or designee) in the event of a medical emergency after discharge from Quarantine.

11.8 Withdrawal Visit (Day 0 to 7)

In the event that a subject withdraws from the study at any time, the reason for discontinuation, if known, must be fully documented in the source documents. The Day 28 (± 5 days) follow up safety assessments may be conducted at the CI or delegate's discretion if the subject agrees.

11.9 Study Follow-up Visit (Day 28 ± 5 days)

The following procedures and assessments will be performed at the Day 28 (± 5 days) Follow-up Visit:

- Adverse events and concomitant medications
- Blood - Biochemistry including Cardiac Enzymes
- Blood – Coagulation
- Blood - Glucose level
- Blood - Haematology
- Blood - HRV-16(RVL) Viral Neutralisation Assay
- Blood - PBMC and RNA
- Body fat, weight, BMI
- Complete physical examination
- Nasal swab ($TCID_{50}$ and/or qPCR)- optional
- Nasal wash ($TCID_{50}$ and/or qPCR)- optional
- Spirometry - optional
- Tympanic temperature
- Urinalysis
- Urine pregnancy test (females only)
- Vital signs: BP, RR, HR and SpO_2
- 12 Lead ECG (optional)
- Breath alcohol test (optional)

This visit marks the completion of the subjects' planned participation in the study.

11.10 Subject Discontinuation (Lost to Follow-up)

If a subject elects not to return to the clinic for their final visit, the CI or delegate should make every effort to contact the subject to review all AEs.

If a subject drops out of the study at any time, the reason, if known, for discontinuation should be fully documented in the source documents; every effort should be made to complete all required withdrawal visit assessments (Section 11.8).

12 DEFINITIONS OF ILLNESS AND INFECTION

12.1 Upper Respiratory Tract Infection

A subject will be considered to have upper respiratory tract infection (URTI) if he/she has any one of the following on two consecutive days, at least one day of which must feature Grade 2 severity, or if any of the following attain Grade 3 severity once, (in conjunction with the physicians Directed Physical Examination) to correlate findings:

- Rhinorrhoea (runny nose)
- Nasal congestion (stuffy nose)
- Sneezing
- Sore throat

12.2 Lower Respiratory Tract Infection

A subject will be considered to have lower respiratory tract infection (LRTI) if he/she has any one of the following on two consecutive days, at least one day of which must attain Grade 2 severity, or if any of the following attain Grade 3 severity once:

- Self-reported symptoms
    - Cough

- Physician findings
    - New wheezes, râles, rhonchi, or other lower respiratory tract signs

12.3 Systemic Respiratory Tract Illness

A subject will be considered to have systemic respiratory tract illness (SRTI) if he/she has any one of the following on two consecutive days, at least one day of which must attain Grade 2 severity, or if any of the following attain Grade 3 severity once:

- Headache persisting for >1 hour
- Myalgia and/or arthralgia
- General malaise
- Chilliness/fever

12.4 Laboratory-Confirmed HRV-16 (RVL) Illness

Laboratory confirmed HRV-16 (RVL) illness is defined as any of the syndromes above, plus infection as defined below:

12.4.1 Laboratory-Confirmed HRV-16 (RVL) Infection

HRV-16 (RVL) Infection will be defined as the presence of viral shedding or Seroconversion.

12.5 Definition of Viral Shedding

A subject will fulfil the criteria for HRV shedding if he/she has a positive cell culture assay at least once during the quarantine post-HRV inoculation; and/or if analysed by qPCR at least 2 positive detections by any qPCR assay, (if single sample, two separate positive aliquots) required between Day one and Day of discharge from Quarantine.

12.6 Definition of Seroconversion

Sero conversion is defined as greater than or equal to a 4 fold increase in antibodies to HRV from baseline.

13 CLINICAL ASSESSMENTS

13.1 Prior and Concomitant Medications

Medications taken up to the time of Screening will be documented at the Screening Visit.

Concomitant medication will be reviewed regularly from Screening until the Follow-up Visit. All new and changed medications will be documented in the source documents.

13.2 Height, Weight, BMI and Body Fat

Height (m), body weight (kg), calculated BMI, and % fat free mass analysis (at the CI or delegate's discretion) will be recorded at Screening.

Body weight, BMI, and % fat free mass analysis will also be calculated at the end of study Follow-up Visit.

BMI will be calculated as BMI [kg/m^2] = Body weight [kg] ÷ Height^2 [m^2]

Details of the analysis of body fat will be documented in the Statistical Analysis Plan (SAP).

13.3 Alcohol Breath Test

At various time points during the study subjects will be asked to breathe into a breath Alco-meter to check for evidence of alcohol consumption.

13.4 Physical Examination

Any clinically significant changes in the physical examination that are not assessed as symptoms of HRV-16 infection during the study will be recorded as an AE and will be dealt with according to RVL's standard procedures. The CI or designee may perform additional physical examination assessments to evaluate or manage clinical illness. Assessments of individual subjects should be made by the same observer wherever possible.

13.4.1 Complete Physical Examination

A complete physical examination will be performed at Screening, Quarantine check-in (Day -2/-1), on discharge, and at the Follow-up Visit.

Complete physical examination includes:

- Examination of the:
  - Cardiovascular system
  - Respiratory system
  - Musculoskeletal system
  - Neurological system
  - Abdominal examination

    - Lymph nodes
    - Skin
- Other general examination as deemed necessary by the CI or delegate

13.4.2 Directed Physical Examination

Directed physical examinations will be conducted according to the appropriate standard procedures as deemed appropriate by the CI or delegate. During the study any clinically significant changes not assessed as symptoms of HRV-16 (RVL) infection will be recorded as AE(s) and dealt with according to RVL's standard procedures.

The directed physical examination includes examination of the:

- Ear
- Nose
- Throat
- Chest (via stethoscope)

Daily (morning) assessment of any upper and lower respiratory symptoms (nasal discharge, otitis, pharyngitis, sinus tenderness, new wheezes, râles and rhonchi) will be performed. Upper respiratory and lower respiratory symptoms will be assessed and graded according to the appropriate standard procedures.

All directed physical examinations assessments will be documented. During the challenge phase, findings thought to be clinically significant i.e. those not due to HRV-16 (RVL) infection, will be captured as AEs (Section 17).

Physician-reported assessments of URTI resulting from challenge virus infection will be graded in accordance with their intensity as absent, mild, moderate or severe and will include:

- Nasal discharge
- Otitis
- Sinus tenderness
- Pharyngitis

13.5 Vital Signs

Vital signs, including BP, RR, HR and SpO_2 will be measured at scheduled times at Screening, throughout the Quarantine period, and at the Follow-up Visit as outlined in Table 2: Time and Events Schedule.

- BP (Systolic and Diastolic) will be recorded after the subject has been sitting for at least 5 minutes.
- Heart rate will be counted for a full minute and recorded in beats per minute (bpm).
- Respirations will be counted for 15 seconds and recorded as breaths per minute.

- SpO_2 will be recorded using a pulse oximeter placed over the end of the subject's finger.

Assignment of severity grading for out of normal range vital signs will be performed according to CTCAE v4.0 guidance. Those deemed to be of clinical relevance will be reported as an AE and will be dealt with accordingly.

The CI or designee may perform additional vital sign or physical examination assessments to evaluate or manage clinical illness. Assessments in any one subject should be made by the same observer wherever possible.

13.6 Temperature

Tympanic temperature will be recorded throughout the study period and at the Follow-up Visit as outlined in Table 2: Time and Events Schedule**.** Measurements taken on Days -2 through to discharge will be evaluated for virulence and pathogenicity of the HRV-16 (RVL) infection.

Temperature is critical to definitions of illness and to symptomatic and specific therapy criteria, therefore temperatures of ≥ 37.9ºC must be confirmed by a repeat measurement not less than 20 minutes and not more than 60 minutes after the first reading. The first temperature measurement will be used if confirmed by the second reading.

13.7 12-Lead Electrocardiogram

12-lead ECGs will be obtained at scheduled times at Screening, on admission to Quarantine, on Day 7 and at the Investigator's discretion at the Day 28 (± 5 days) Follow-up Visit as outlined in Table 2: Time and Events Schedule**.** Any clinically significant changes from Screening will be recorded as an AE and dealt with according to RVL's standard procedures.

The following data will be automatically calculated:

- Heart rate (HR) (normal 50-100 beats per minute (bpm))
- PR interval (normal 0.12 to 0.2 sec)
- QRS duration (normal < 0.12 sec)
- QT interval (normal < 0.33-0.44 sec)
- QTc interval (normal- male <0.43 sec, female < 0.45 sec)

13.8 Spirometry

Spirometry will be conducted according to RVL standard procedures. Any clinically significant change in pulmonary function during the study will be recorded as an AE.

Spirometry will be performed at Screening, on admission to Quarantine, at the Physician's discretion during the Quarantine period, and at the Follow-up Visit. Three technically-acceptable measurements should be made at each time-point and recorded in the source documents. The best reading from each assessment will be used for analysis.

The following will be calculated:

- FEV_1 % predicted
- FEV_1/FVC ratio (absolute, not predicted)
- FEF $_{25\%-75\%}$

13.9 Tissue Count and Mucus Weight

If appropriate, all paper tissues used by each subject will be collected for each 24-hour period throughout the Quarantine period, to determine number of tissues used and the weight of mucus expelled.

Paper tissue and bag distribution will start on Day -1 at approximately 0800 (± 1h).

Subjects will be given pre-weighed packets of paper tissues. After a tissue has been used for nose-blowing or sneezing into, the subjects should place them into separate bags for collection the following morning.

Collection of used tissues will start on Day 0 at approximately 0800 (± 1h) and will be performed daily until Day 8 or until the subject is finally discharged.

13.10 Symptom Diary Card

Subject symptoms will be recorded on the RVL Symptom Diary Card at scheduled times from Day -2 throughout the Quarantine period, as outlined in Table 2: Time and Events Schedule. Self-reported assessments of illness resulting from HRV challenge virus infection are described in Section 12.1.

Any other symptoms noted by the subject will also be recorded. This information will be entered into the source documents by the subjects via the symptom diary card or by non-leading clinician questioning. Findings greater than Grade 0 will be presumed to represent HRV-16 (RVL) infection consequent to challenge, and will not be additionally captured as AEs (Section 17) unless they meet one of the criteria for a SAE (Section 17.1).

The CI will review the subject's symptom diary card entries on a daily basis after admission to the Quarantine Unit. Entries considered to be AEs will be entered in the source documents.

13.11 Adverse Events

The CI or delegate is responsible for the detection and documentation of events meeting the criteria and definition of an AE or SAE (Section 17) from the time written informed consent is obtained until completion of the Follow-up Visit.

Subjects will be asked to report any unusual, undesirable, or unwanted symptoms, worsening of existing conditions or changes in their health and wellbeing throughout the study from Screening to the Follow-up Visit.

After the subject has had an opportunity to spontaneously report any problems, the CI or delegate will periodically enquire about the occurrence of AEs and medications. During Quarantine, subjects will be asked about AEs twice a day.

The following are examples of open-ended questions that may be used to obtain this information:

- How are you feeling?
- Have you had any medical problems since your last visit/assessment?
- Have you taken any new medicines, other than those given to you in this study, since your last visit/assessment?

14 LABORATORY SAFETY ASSESSMENTS

All abnormal laboratory values will be evaluated by the CI or delegate for clinical relevance and Version 4 of the Common Terminology Criteria for Adverse Events (CTCAE) grading. Those deemed to be of clinical relevance will be reported as defined in Section 17. All abnormal laboratory safety parameters should be flagged with the clinical relevance on the laboratory reports and signed by the investigator according to RVL SOP's.

14.1 Blood

14.1.1 Haematology and Biochemistry

Up to but not in excess of 473 mL of blood will be drawn during the study.

Blood samples for Haematology and Biochemistry will be collected at Screening, on entry to Quarantine, on Days 3 and 7, and at the end of study Follow-up Visit on Day 28 (± 5 days). Additional samples may be taken if required at the discretion of the CI or delegate.

Samples will be analysed for the following parameters:

<u>Haematology</u>

- Platelet Count
- WBC Differential
- Red blood cell (RBC) Count
- Neutrophils
- WBC (absolute)
- Lymphocytes
- Reticulocyte Count
- Monocytes
- Haemoglobin
- Eosinophil's
- Haematocrit
- Basophils
- Mean corpuscular volume (MCV)
- Mean corpuscular haemoglobin (MCH)
- Mean corpuscular haemoglobin concentration (MCHC)

<u>Clinical Biochemistry</u>

- Total and direct bilirubin
- Fasting Glucose (only if clinically indicated at CI's discretion)
- Lactate dehydrogenase (LDH)
- Sodium

- Gamma glutamyl transferase (GGT)
- Potassium
- Albumin
- Chloride
- Alkaline phosphatase (ALP)
- Bicarbonate
- Aspartate aminotransferase (AST)
- Calcium
- Alanine aminotransferase (ALT)
- Urea
- TFT
- Uric Acid
- Total Protein
- Creatinine
- C-reactive protein (CRP)

14.1.2 Coagulation

Blood samples for Coagulation studies will be collected at Screening, on entry to the Quarantine Unit, on Days 3 and 7, and at the end of study Follow-up Visit on Day 28 (± 5 days). Additional samples may be taken if required at the discretion of the CI or delegate.

- Prothrombin time (PT)
- Activated partial thromboplastin time (APTT)
- International normalised ratio (INR)
- APTT Ratio

14.1.3 Cardiac Enzymes

Blood samples for cardiac enzymes will be collected at Screening, on entry to the Quarantine Unit, on Day 0 pre-inoculation (at the CI's discretion) and at the end of study Follow-up Visit on Day 28 (± 5 days). Additional samples may be taken if required at the discretion of the CI or delegate.

- Troponin I/T

14.1.4 HIV, Hepatitis B and C

Blood samples for Human Immunodeficiency Virus (HIV), HBV and HCV antibodies will be collected at Screening.

14.1.5 Blood Glucose

Blood samples will be used to determine blood glucose levels.

14.1.6 Pregnancy Test

A blood serum β-HCG pregnancy test may be performed at the CI or delegate's discretion on Day -2/ Day -1.

14.2 Urine

14.2.1 Urinalysis

Urinalysis will be performed using dipsticks at Screening, Day -2 or Day -1, and Days 3, 7 and 28.

- pH
- ketones
- Protein
- nitrite
- Bilirubin
- Urobilinogen
- Blood
- Leukocytes
- Glucose
- Specific gravity

14.2.2 Class A Drugs and Cotinine Screen

To check for the presence of Class A drugs of abuse and nicotine during the study, urine samples will be collected at various time points and will be tested using the RVL standard test kit. Samples will be collected at Screening, on Day -2/ -1 and at the Physician's discretion during the Quarantine period.

14.2.3 Pregnancy test

All females will have a pregnancy test at the Screening visit, on Day -2/Day -1, Day 0 (pre-inoculation), Day 8, and at the end of study Follow-up Visit (Day 28 ± 5 days). Urine pregnancy tests may also be performed at any time during the study at the CI or delegate's discretion.

15 SAMPLES FOR VIRAL INFECTIVITY

15.1 Throat Swab

Throat swabs will be collected on Day -2; and for $TCID_{50}$ and/or qPCR on Day 3, Day 7, and Day 8. This procedure will be performed as per RVL SOPs.

Throat swabs will always be performed prior to nasal wash procedures.

A throat swab will be performed at Screening for tolerance to the procedure.

15.2 Nasal Swab

A nasal swab will be performed at Screening for tolerance to the procedure.

A nasal swab for Respiratory Virus Screen (DFA) will be performed as per RVL operating instructions on entry to Quarantine (Day -2/Day -1).

Nasal swabs for $TCID_{50}$ and/or qPCR will be performed three times daily (approximately 8 hourly) on Days 1 to 7 of Quarantine, on Day 8 prior to discharge and an optional sample may be taken at the study Follow-up Visit on Day 28 (± 5 days).

Nasal swabs will always be performed prior to nasal wash procedures.

15.3 Nasal Brush

Nasal brush is optional and, if the subject consents, may be undertaken at the CI or delegate's discretion at Screening (to determine tolerance), on admission to the Quarantine Unit (Day -2 only), and on Day 8 prior to discharge from the Quarantine Unit.

15.4 Nasal Scrape

Nasal scrape is optional and, if the subject consents, a nasal scrape may be undertaken at the CI or delegate's discretion at Screening (to determine tolerance), on admission to the Quarantine Unit (Day -2 only), and on Day 8 prior to discharge from the Quarantine Unit.

15.5 Nasal Wash

A nasal wash will be performed according to RVL's SOPs at the Screening visit (to determine tolerance), on admission to the Quarantine Unit (Day -2/ Day -1), twice daily during Days 1 to 7 of the Quarantine period, on Day 8 prior to discharge, and an optional sample may be taken on Day 28 (± 5 days).

The nasal wash should always be performed after a nasal swab.

15.6 Exhaled Breath Condensate

Exhaled breath condensate collection is optional and if the subject consents to the procedure, may be performed at the CI or delegate's discretion on Day 0 pre and post- HRV-16 (RVL) challenge, and daily on Days 1 to 8 of the Quarantine period.

16 SAMPLE ANALYSIS

The following methods of analyses are detailed in the AP. Samples will be collected as described in Table 2: Time and Events Schedule.

16.1 HRV-16 (RVL) Neutralisation Assay

To be eligible for the study a subject must be 'sero-suitable' i.e. have low immunity to the Challenge Virus (HRV-16 (RVL)) as defined by low-neutralising antibodies to HRV-16 (RVL). Each subject will be initially screened for eligibility based on pre-existing HRV specific antibody responses to HRV-16 (RVL) using a virus neutralisation assay as described in the AP.

16.2 Direct Fluorescence Antibody – (DFA) - Respiratory Viral Panel

Analysis of nasopharyngeal swab samples will include DFA assay: to detect respiratory viruses that could potentially contraindicate a volunteer's participation in the study, samples will be obtained at Screening and on Day -2/ Day-1.

16.3 Quantitative Polymerase Chain Reaction (qPCR)

Analysis of nasopharyngeal samples will include HRV-16 (RVL) viral load as indicated by the time and events schedule. Viral load will be analysed using quantitative polymerase chain reaction (qPCR).

16.4 Inflammatory Biomarkers (PBMC)

Inflammatory biomarker assessments (cytokines) and viable cell counts will be performed on nasal wash samples.

Analysis of cytokines may be performed on blood samples.

16.4.1 Humoral Immunity

Humoral immunity may be analysed using suitable assays such as a virus neutralisation assay or an optional enzyme-linked immuno-sorbant assay (ELISA). ELISA is a biochemical technique used to detect the presence of an antibody or an antigen.

16.4.2 Cell-Mediated Immunity

Uncoagulated whole blood will be collected and sent immediately to an appropriate laboratory for the isolation and cryopreservation of peripheral blood mononuclear cells (PBMC). Cryopreserved PBMC will be tested for interferon (IFN) and to determine aspects of T cell immunity.

16.4.3 Viral RNA

16.4.3.1 Micro RNAs (PAXgene)

This optional assay may be performed. MicroRNAs (small ~22 nucleotide long non-coding endogenous RNAs) are critical regulators of gene expression in multi-cellular eukaryotes. Recent studies have proved that viruses also express microRNAs, which are thought to contribute to the intricate mechanisms of host-pathogen interactions [4].

The microRNA pool will be isolated from whole human blood collected using PAXgene™ RNA tubes (PreAnalytiX, Valencia, CA), or similar, and quantitated on a PCR-based array platform.

16.4.3.2 Messenger RNA Expression

This optional assay may be performed. Messenger RNA (mRNA) will be extracted from PBMCs using standard techniques and analysed using micro array technology. In standard microarrays, the probes are synthesized and then attached via surface engineering to a solid surface by a covalent bond to a chemical matrix (via epoxy-silane, amino-silane, lysine, polyacrylamide or others).

16.4.4 Tissue Culture Infective Titre ($TCID_{50}$)

This endpoint dilution assay quantifies the amount of virus required to cause a pathological change in 50% of the cell-containing wells inoculated. Host cells are plated onto suitable 96 microwell plates, the cells are allowed to adhere and reach a suitable level of confluency, and serial dilutions of the virus are then added. After incubation, the percentage of cell death (i.e. infected cells) measured for each virus dilution, and results are used to mathematically calculate a $TCID_{50}$ result [12].

17 ADVERSE EVENTS

An AE is any untoward medical occurrence in a subject, regardless of its relationship to the product being studied/ administered.

An AE can be any unfavourable and unintended sign (including an abnormal laboratory finding), symptom, or disease temporally associated with the use of the challenge virus, whether or not considered related to the challenge virus.

An AE includes:

- Exacerbation of a pre-existing illness
- Increase in frequency or severity of a pre-existing episodic condition
- A condition detected or diagnosed after challenge virus administration even though it may have been present prior to the start of the study
- Complications and termination of a pregnancy

An AE does not include:

- Medical or surgical procedures (e.g. surgery, endoscopy, tooth extraction, transfusion); the condition that leads to the procedure is an AE
- Pre-existing disease or conditions present or detected prior to start of challenge virus administration that do not worsen
- Hospitalisation for elective surgery, social and/or convenience admissions provided they are arranged before the start of challenge virus administration
- Over- administration of either virus inoculum or concomitant medication without any signs or symptoms
- An uncomplicated pregnancy or an induced elective abortion to terminate a pregnancy without medical reason

All AEs and SAEs will be recorded in the source documents and will include the nature, date and time of onset, intensity, duration, causality, and outcome of the event. Even if the AE is assessed by the CI or delegate as not reasonably attributable to the challenge virus, its occurrence must be recorded in the source documents.

17.1 Serious Adverse Events

A SAE is an untoward medical occurrence that:

- Results in death
- Is life-threatening
- Requires inpatient hospitalisation or prolongation of existing hospitalisation
- Results in persistent or significant disability/incapacity

- Is a congenital anomaly/birth defect

The term life-threatening refers to an event in which the patient was, in the opinion of the CI or delegate, at risk of death at the time of the event. It does not refer to an event which hypothetically might have caused death if it were more severe.

Medical and scientific judgment should be exercised in deciding whether expedited reporting is appropriate in other situations, such as important medical events that may not be immediately life-threatening or result in death, but may jeopardize the patient or may require intervention to prevent one of the outcomes listed in this definition above. These should also usually be considered serious.

Examples of such events are intensive treatment in an acute care environment or at home for allergic bronchospasm, blood abnormalities or convulsions that do not result in inpatient hospitalisation, or the development of drug dependency or drug abuse.

17.2 Adverse Drug Reaction

An Adverse Drug Reaction (ADR) is any untoward and unintended response in a subject to a medicinal product which is related to any dose administered to that subject.

This study is a Human Viral Challenge study and no IMP is given. Adverse drug reactions are therefore unlikely unless related to the administration of concomitant medications.

17.3 Clinical Laboratory Abnormalities and Other Abnormal Assessments

Laboratory and other safety assessments will be reviewed by a Physician for any values outside the expected reference range and documented as an AE if they should meet the criteria laid down in the CTCAE v 4.0.

Any abnormalities will be recorded as either clinically significant (CS) or not clinically significant (NCS). All AEs should be captured on the AE log regardless of clinical significance. In some cases, significant changes within the normal range may require similar judgment by the CI or delegate.

Laboratory abnormalities which are deemed clinically significant will be re-assessed by the CI or delegate. For example, after a period of monitoring a subject’s elevated transaminase results, the CI or delegate may deem the clinically significant elevation to have changed the AE.

All clinically significant laboratory abnormalities deemed to be AEs or SAEs should be listed on the AE page of the source documents, and causality to challenge virus should be assessed.

All significant abnormal laboratory results or assessments will be followed until they resolve (i.e. return to normal or baseline values), stabilise, or until they are judged by the CI or delegate to be no longer clinically significant.

Follow-up continuing beyond Day 28 may be required. The CI or delegate may refer to the subject to his/her GP or other healthcare professional for follow-up, as appropriate.

All abnormal laboratory values of any causality (clinically or not clinically significant) may be repeated or monitored as necessary.

17.4 Recording of Adverse Events and Serious Adverse Events

All AEs, with the exception of spirometry, will be recorded from the Screening baseline visit until the Day 28 Follow-up Visit, or until the resolution of the AE. They must be fully recorded in the source documents as they are reported, whether spontaneously volunteered by a subject or in response to questioning about wellbeing at each study visit. Enquiries about AEs should cover the period between the previous and current visit.

Any drop in spirometry pre-inoculation will not be due to the Challenge Virus so will not be considered as an AE unless there are other factors to consider which may make it clinically significant. Therefore, the baseline for Spirometry will be Day-1 visit for all subjects.

- The CI or delegate should review all documentation (e.g. laboratory, or diagnostic reports) relative to the event being reported, and record all relevant information in the source documents.
- The CI or delegate will assess each AE as follows:

17.4.1 Description

If the event consists of a cluster of signs and symptoms, a diagnosis should be recorded [e.g. gastroenteritis] rather than each sign and symptom.

17.4.2 Onset and End

The dates and times of the onset and end of the event should be recorded.

17.4.3 Intensity and Severity

Intensity is defined as one of the following:

- Mild: awareness of sign or symptom, but easily tolerated
- Moderate: discomfort sufficient to cause interference with normal activities
- Severe: incapacitating, with inability to perform normal activities

It is important to distinguish between serious and severe AEs. Severity is a measure of intensity whereas seriousness is defined by the criteria provided in Section 17.1. An AE of severe intensity need not necessarily be considered serious. For example, a migraine headache that incapacitates a subject for many hours may be considered a severe AE, whereas a stroke that

results in a limited degree of disability may be considered mild, but should be reported as an SAE.

17.4.4 Seriousness

The CI or delegate must record whether or not the AE meets the definition of serious. If the event is serious, the CI or delegate must complete a SAE Report Form (see definition of SAEs Section 17.1).

17.4.5 Relationship or Causality

Every effort should be made by the CI or delegate to assess the relationship of the AE, if any, to the Challenge Virus; any study procedure or assessment; any concomitant medication or treatment. Causality should be classified using the categories presented below.

Table 3: Classification of AE Causality

Classification	Definition
Related	There is an association between the event and the administration of Challenge Virus Inoculum, a plausible mechanism for the event to be related to the Challenge Virus Inoculum and causes other than the Challenge Virus Inoculum have been ruled out, and/or the event re-appeared on re-exposure to the Challenge Virus Inoculum.
Possibly Related	There is an association between the event and the administration of the Challenge Virus Inoculum and there is a plausible mechanism for the event to be related to Challenge Virus Inoculum, but there may also be alternative aetiology, such as characteristics of the subject's clinical status or underlying disease.
Unlikely Related	The event is unlikely to be related to the Challenge Virus Inoculum and likely to be related to factors other than Challenge Virus Inoculum.
Not Related	The event is related to aetiology other than the Challenge Virus Inoculum (the alternative aetiology must be documented in the study subject's medical record).

17.4.6 Outcome

An AE should be followed until the CI or delegate has determined and provided the final outcome or an alternative explanation has been provided. The outcome should be classified according to the categories shown in Table 4.

Table 4: Classification of AE Outcome

**Classification**	**Definition**
Recovered/Resolved	Resolution of an AE with no residual signs or symptoms
Recovered/ Resolved With Sequelae	Resolution of an AE with residual signs or symptoms
Not Recovered/ Not Resolved (Continuing)	Either incomplete improvement or no improvement of an AE, such that it remains on-going
Fatal	Outcome of an AE is death. “Fatal” should be used when death is at least possibly related to the AE.
Unknown	Outcome of an AE is not known (e.g., a subject lost to follow-up)

17.4.7 Action Taken

The following actions are possible:

- None
- AE required treatment

All applicable actions taken should be recorded.

17.4.8 Follow-up

All AEs and SAEs must be followed-up by the CI or delegate or referred to the subject’s GP for follow-up until they are resolved (return to normal or baseline values) or stabilised, or until they are judged by the CI or delegate to be no longer clinically significant. Supplemental measurements and/or evaluations may be necessary to fully investigate the nature and/or causality of an AE or SAE. This may include additional laboratory tests, diagnostic procedures, or consultation with other healthcare professionals. If the subject dies, any post-mortem findings (including histopathology) must be provided to RVL.

17.4.9 Pregnancy

Pregnancy of a study subject or subject’s partner during or within 3 months of inoculation is not a SAE but must still be reported to the CI in all instances. However, as congenital anomaly/birth

defect are SAEs, on-study pregnancies must be recorded to allow follow up to birth. Information related to the pregnancy must be reported as per the RVL SOP. The pregnant individual may be referred to their GP or to a specialist, as appropriate. The CI will be responsible for informing the REC as appropriate.

17.5 Reporting SAEs

All serious adverse events (SAEs) should be reported immediately to the CI and sponsor (Medical Expert). Immediate and follow up reports will be completed promptly and as per RVL SOPs These reports will identify subjects by a unique code number assigned to the subject rather than by the subject's names, personal identification numbers and or addresses

**Reporting SAEs**	
**Contact Person:**	Dr Ganesh Balaratnam
**Telephone Number**	0845 330 5664
**e-mail address**	RVLSAEreporting@retroscreen.com

There are no SAEs identified that would not require immediate reporting, Grade 3 to 4 laboratory abnormality must be reported to the CI or delegate, who will determine if appropriate to report immediately to the sponsor (Medical Expert).

The CI in collaboration with the Sponsor (Medical Expert) should promptly notify the REC as per RVL SOPs of all findings:

- That could adversely affect the safety of subjects,
- Impact the conduct of the trial
- Alter the ethics favourable opinion to continue or are
- Fatal or life/threatening

<table>
<tr><th rowspan="2">Type of SAE</th><th colspan="2">INITIAL REPORTS</th><th>FOLLOW-UP REPORTS</th></tr>
<tr><td>Fatal or
Life-threatening SAE</td><td>Other SAEs</td><td>Any SAE</td></tr>
<tr><th rowspan="2">REPORTING REQUIREMENTS</th><td>Within 24 hours

Telephone notification</td><td rowspan="2">Within 48 hours

Fully completed SAE Report Form to RVL Medical expert</td><td rowspan="2">Within 48 hours

Updated SAE Report Form to RVL Medical expert</td></tr>
<tr><td>Within 48 hours

Fully completed SAE Report Form to RVL Medical expert</td></tr>
</table>

17.5.1 Research Ethics Committee

The CI or delegate must notify the REC of any serious or unexpected AEs regardless of relationship to the Challenge Virus. Concurrently, the CI must send documentation of notification of the REC to the RVL Medical expert. The REC will be sent 6-monthly safety updates in order to facilitate their continuing review of the study as required.

17.6 Post-Study Obligations

CI or delegates are not obligated to actively seek AEs or SAEs in former study participants. However, the CI or delegate should promptly notify the CI who will in turn notify the RVL Medical expert if he learns of any SAE or death of a study subject within 30 days after a subject has been discontinued from the study, and such event(s) is (are) reasonably related to the Human Viral Challenge.

18 STATISTICAL METHODS AND PLANNED ANALYSES

S-cubed on behalf of RVL will perform statistical analysis for the study.

18.1 Study Populations

The safety population is defined as all subjects receiving HRV-16 challenge virus inoculum; this population will be used in presenting baseline and safety data. The primary analysis will be based on the safety population.

The virulence and pathogenicity population is defined as all sero-suitable subjects receiving HRV-16 challenge virus inoculum. The Per Protocol (PP) population is defined as all virulence and pathogenicity population subjects who have no major protocol deviations and who complete the Quarantine period up to the final day of Quarantine, Day 8 (Discharge).

In terms of the virulence and pathogenicity analysis, the primary concern will be the virulence and pathogenicity population, with the PP population being of secondary concern. All virulence and pathogenicity analyses will be performed on both of these patient populations.

In all reporting presentations where quarantine group is shown, subjects will be grouped and presented against by the titre of challenge virus that the subject actually received (Group A: 1 x$TCID_{50}$, Group B: 10 x $TCID_{50}$, Group C: 100 x $TCID_{50}$).

18.2 Data Management

The Sponsor (RVL) will be responsible for the supervision of the overall conduct of data management for this study, using qualified individuals throughout all stages in accordance with the principles of GCP.

- Trial process from design
- eCRFs data handling
- Data verification
- Planning and conducting the statistical analyses (in conjunction with S-cubed)
- Preparing the final report

Prior to commencing data processing, a Data Management Plan (DMP) will be produced, which will describe and define all data management activities, and the database structure and contents.

Data will only be collected from subjects that have signed a written ICF and in accordance with the DMP via the eCRF provided by SureSource^{TM}. RVL will ensure in conjunction with the eCRF provider this electronic data processing system conforms to the established requirements for

- Completeness
- Accuracy

- Reliability
- Consistency with intended performance (i.e. Validation)
- Maintain SOPs for use
- Ensure the system is designed to permit data changes to maintain an audit trail, data trail and edit trail
- Maintain a security system the prevents unauthorised access to the data
- Maintain a list of the individual authorised to make data changes
- Safeguard the blinding
- It will be possible to compare the original data and observations if the data is processed
- An unambiguous subject identification code that allows identification of the all the data reported will be used.

Prior to database lock the database will undergo quality control as detailed in the DMP. A 100% check will be carried out on critical variables. Acceptance rates for critical variables will be 0% error, and 0.25% for all other data. Following database lock, the final database will be transferred as SAS™ datasets to the Study Statistician for statistical analysis and reporting as per the agreed SAP.

Data privacy regulations in the UK will be complied with by the study site. All source documents must be kept in order and up-to-date so that they reflect the latest observations on the enrolled subjects. All entries, corrections, and alterations must be made by the CI or delegate or other authorised study-site personnel. The CI or delegate must verify that all data entries in the source documents are accurate and correct.

Source data will be collected either in paper form or directly in to the eCRF (SureSource) as documented in the Source Document agreement.

During the study, data will be made available for review by the Sponsor, CI or delegate (CI or designee), and statistical review via pre-defined reports extracted from the database at agreed intervals documented in the DMP.

18.3 Data Coding

AEs and concomitant medications will be coded as documented in the DMP.

18.4 Sample Size

No formal sample size calculation has been performed for this Phase I study.

Eighteen subjects will be studied in 3 groups of 6 subjects each. Subjects will be enrolled into each of the following 3 groups:

- Group A: 1 x $TCID_{50}$
- Group B: 10 x $TCID_{50}$
- Group C: 100 x $TCID_{50}$

Additional subjects will be available to act as reserves if required.

18.5 Interim Analysis

No formal statistical interim analysis of data will be performed.

18.6 Randomisation

This is a randomised study. Subjects will be randomly allocated to either quarantine groups A, B or C pre inoculum via a randomisation code list. The randomisation code list will be computer generated using a permuted block algorithm in a 1:1:1 ratio for the three quarantine groups.

18.7 Statistical and Analytical Plan

Data will be analysed and reported using SAS® version 9.2 or a later version.

Summary tables will be presented by quarantine group and also for demographic and baseline data, for all subjects.

Due to the size of the patient population in this study no statistical comparison of groups are planned to be performed. The study will be summarised in terms of descriptive statistics. Continuous variables will be summarised using number of observations, mean, standard deviation, median, minimum and maximum values. Categorical variables will be summarised using proportions (counts and percentages).

A detailed Statistical Analysis Plan (SAP) will be developed and approved by RVL prior to any lock of the study database. The SAP will give a more detailed description of the report presentations to be produced, expanding on the protocol specified analysis. Any deviation from the protocol specified analysis will be documented within a protocol or SAP amendment, as appropriate, and described within the CSR. Further post-hoc evaluations of exploratory endpoints may be performed and these will be separately reported and identified in the CSR.

18.7.1 Subject Accountability

The number of subjects enrolled, withdrawing (also split by reason for withdrawal) from and completing the study, and the number in each analysis population will be summarised for all subjects and by group. Accounting for the occurrence of and extent of missing data, and its possible impact on the study analysis will be described within the SAP.

18.7.2 Protocol Deviations

Subject data will be reviewed for major protocol deviations prior to database lock at a planned data review meeting, and decisions will be documented within the meeting minutes. At this meeting the analysis populations will also be reviewed and finalised.

18.7.3 Subgroup Analysis

No sub-group analyses are planned.

18.7.4 Demographic and Baseline Characteristics

Descriptive statistics of demographics (Age, Sex, Height, Weight, BMI, body fat and Ethnicity at Screening) will be presented by group and overall for the safety population. Medical history information will be listed.

18.7.5 Virulence and Pathogenicity Analysis

The primary endpoint is the AUC of HRV-16 (RVL) virus shedding, which will be summarised by group.

Descriptive statistics will be presented for the following other secondary endpoints by group:

- Humoral and cell mediated immunity
- % of subjects infected with HRV-16 (RVL)
- AUC of symptoms
- Severity of symptoms
- % of subjects with fever
- % of subjects with:
    - URTI
    - LRTI
    - SRTI
    - Any illness
- % of subjects with Grade 2 or worse symptoms
- Immune responses

18.7.6 Safety Analysis

18.7.6.1 Adverse Events

The safety endpoint of the study is the incidence of virus challenge emergent AEs which are not consistent with a mild to moderate common cold infection, which will be summarised by group.

In addition, the incidence of all virus challenge emergent AEs will be reported within summary presentations, by MedDRA system organ class and preferred term, by group. An AE will be classified as virus challenge emergent if the onset date of the AE is on or after the date of inoculation (Study Day 0). Should any onset date for an AE be missing or only a partial date

recorded (such that it cannot be determined if the event onset was prior to virus inoculation or not) then it will be assumed that the event is virus challenge emergent, unless the AE stop date indicates otherwise.

If a subject experiences more than one AE with the same preferred term, that preferred term will be counted only once in summary presentations. It will be assigned the greatest observed severity and the strongest relationship to the challenge virus among those events for summaries in which those characteristics are considered.

Summary presentations will be produced for the number and percentage of subjects reporting virus-emergent: AEs, severity of AEs, AEs by relationship to challenge virus, SAEs, severity of SAEs, and SAEs by relationship to the challenge virus. In addition SAEs and AEs directly resulting in withdrawal from study will be listed.

18.7.6.2 Laboratory Parameters

Laboratory parameters (Haematology, Clinical Biochemistry, Coagulation, Cardiac Enzymes, Blood Glucose, and Urinalysis) will be included in subject listings. Laboratory values outside the normal range will be identified in these subject listings.

18.7.6.3 Vital Signs

Vital signs parameters (SBP, DBP, RR, HR and $SpO_{2)}$ will be included in subject listings.

18.7.6.4 Physical Examination

Physical examination findings (both for complete and directed examinations) will be included within subject listings.

18.7.6.5 Spirometry

Spirometry parameters (FEV_1 % predicted, FEV_1/FVC ratio (absolute), and $FEF_{25\%-75\%}$) will be included in subject listings.

18.7.6.6 12-Lead ECG

ECG parameters (HR, PR, QRS, QT and QTc) will be included within subject listings.

18.7.6.7 Concurrent Medications

Concomitant medications will be coded to their generic name and Drug Class (L2) using the latest version of the WHO dictionary. Medications will be assigned as being prior to or concomitant with challenge virus (i.e. given prior to or after receiving challenge virus) based on the start and stop dates of the medication and the timing of receiving the challenge virus. If the medication stop date is before the date of virus inoculation, the medication will be assigned as being prior to challenge virus. In all other situations, the medication will be assigned as being

concomitant with the challenge virus. Prior and concomitant medications (separately identified) will be included in subject listings.

19 STUDY MANAGEMENT AND RESPONSIBILITIES

Before the first subject is enrolled in the study, all ethical and legal requirements must be met.

19.1 Ethics Review

The protocol and ICF will be reviewed and approved by a properly-constituted REC.

19.2 Regulatory Approval and Good Clinical Practice

This study will be conducted in accordance with the ICH GCP; the Declaration of Helsinki (1996) and the REC approved study protocol.

19.3 Protocol Deviations

Any unplanned or unintended departures from the study protocol will be documented on a protocol deviation log as soon as possible after they occur, by the CI, or other appropriately qualified person.

19.3.1 Serious Breach

Any serious breach of the conditions and principles of GCP in connection with the study will be reported to the Sponsor and the REC. A serious breach is defined as any breach that is likely to effect to a significant degree either:

- The safety or physical or mental integrity of the subjects
- The scientific value of the trial

19.4 Protocol Amendments

Neither the CI or delegate nor RVL will alter the study protocol without obtaining the written agreement of the other party. Once the study has started, amendments should be made only in exceptional cases. The changes then become part of the study protocol.

Amendments can be divided into substantial and non-substantial amendments. For all amendments an evaluation will be made by the CI as to whether an amendment meets the definition of substantial i.e. it is likely to have a significant impact on any one of the following:

- Safety or physical or mental integrity of the subjects
- Scientific value of the trial
- Conduct or management of the trial
- Quality or safety of the IMP used in the trial (not applicable in this study)

The CI must approve all amendments before implementation. Amendments that have an impact on subject risk or the clinical trial objectives, or require revision of the informed consent document, must receive approval from RVL and the REC prior to their implementation.

When a protocol amendment substantially alters the clinical trial design or the potential risks or burden to subjects, the ICF will be amended and approved by the REC and all active subjects will be asked to reconfirm their continued willingness to participate in the trial.

A log of all protocol amendments, together with their designation as substantial or non-substantial, will be maintained to support clinical study reporting.

19.5 Discontinuation of the Study

RVL reserves the right to temporarily suspend or discontinue the study for any reason. In addition, the study may be stopped at any time if, in the opinion of the RVL Medical Expert safety data suggest that the medical safety of subjects is being compromised.

RVL reserves the right to terminate the study for refusal of the CI or delegate to supply source documentation of work performed in this clinical study. In such a case, the CI or delegate is responsible for informing the REC of the study termination.

If the study is suspended or terminated for safety reason(s), the CI will promptly inform the REC of the suspension or termination of the study and the reason(s) for the action.

If the study is prematurely terminated, all study data must be returned to RVL. The site must also conduct final disposition of all unused protocol mandated non-IMP in accordance with RVL SOP – CLIN-010.04 Management of Non IMP and Concomitant Medication.

19.6 Study Records Retention and Direct Access to Source Documents

The CI or delegate shall keep a copy of the paper and electronic source documents and the CI or delegate's Site File (ISF). Data will be reviewed and signed by the CI or delegate, or designee as applicable.

The CI or delegate agrees to allow inspections of the study site and any source documentation by clinical research and audit personnel, external auditors or representatives of regulatory authorities/ethics committees, and will allow direct access to source documents.

Direct access to the subject's paper or electronic medical/clinical records (if applicable to the study) is necessary to verify and corroborate the data recorded on the source documents. This procedure is termed Source Document Verification (SDV). During the review of these documents, the anonymity of the subject will be respected. The CI or delegate shall keep the paper and electronic source documents as per RVL SOPs.

19.7 Sponsor Responsibilities

It is the Sponsor's responsibility to obtain the appropriate approvals to perform the study, and to report to regulatory authorities (if appropriate) the results of this study.

19.8 Monitoring and Auditing

The Sponsor will determine the appropriate extent and nature of the monitoring, to verify that the as documented in the Monitoring plan

- The rights and wellbeing of the subjects are protected.
- The reported trial data are accurate, complete and verifiable from source.
- The conduct of the study is in compliance with the approved protocol and GCP.

The CI will allow the Study Monitors direct access to all relevant documents, and allocate his time and staff to the Monitor to discuss and resolve findings or relevant issues.

19.9 CI or Delegate Responsibilities

19.9.1 Ethical Considerations

The CI or delegate will make all required REC submissions and notifications/updates and obtain all required approvals in accordance with RVL SOP's.

Appropriate reports on the progress of the study will be made to the REC and the Sponsor by the CI or delegate in accordance with local regulatory practices and in agreement with the Sponsor.

The REC will be informed about the end of the trial with the required timelines. In the event of early termination this is 15 days from trial completion and the reason for termination is required. In the case of routine termination, this is 90 days from the protocol-defined end of trial date or event.

19.9.2 Informed Consent

Before entering the subject into the study the study procedures and any known or likely risks will be explained to the subjects in lay language by the CI or delegate or designated physician and any questions will be answered. A properly executed, written, informed consent form (ICF), in compliance with the Declaration of Helsinki, ICH GCP, and other applicable regulations will be obtained from subjects willing to participate.

The ICF must be signed and dated by the subject and countersigned by the CI or delegate or designated physician (whoever conducted the consent discussion). All subjects will receive a copy of the signed ICF, as well as a written participant/volunteer information sheet. The CI or delegate will retain the original ICF in the Site File with the Subject Screening and Subject Enrolment Logs to be checked by the monitor during monitoring.

The subjects will be assured that they can withdraw from the study at any time and for any reason without prejudice to their medical care. The subjects will also be informed in a timely manner if new information becomes available that may affect their willingness to continue participation in the study. The communication of this information must be documented.

If the ICF is amended during the study, the CI or delegate must follow all applicable regulatory requirements pertaining to approval of the amended ICF by the REC. The site must use the amended ICF for all new subjects and repeat the consent process with the amended ICF for any on-going subjects, if required.

19.10 Laboratory Certification and Normal Values

The CI or delegate will provide the name and location of the clinical laboratory (s) used for laboratory tests, retain a copy of certification for all laboratory tests included in the protocol, certification number, date of certification, and a list of the normal values for all laboratory tests required by the protocol. These documents must be available prior to any subject being treated in the study. Updated versions of these documents must be provided as appropriate.

19.11 Delegation of CI or delegate Responsibilities

The CI or delegate should ensure that all persons assisting with the study are adequately informed about the protocol, any amendments to the protocol, the study medication, and their study-related duties and functions. The CI or delegate should maintain a list of sub-CI or delegates and other appropriately qualified persons to whom he or she has delegated significant study-related duties.

19.12 Information for Subject’s General Practitioner

The subject’s GP will be advised by letter of the subject’s participation in the study.

19.13 Compensation and Expenses

Subjects will be reimbursed for their inconvenience and out-of-pocket expenses, including travelling costs. All proposed payments to subjects will be approved by the REC prior to the start of the study and the amount of payment will be specified in the Subject Information Sheet.

19.14 Non-Protocol Research

No investigational research procedures pertaining to this study other than those outlined in this protocol may be undertaken on the subjects or their biological samples without the prior written permission of the subject, the Sponsor, the REC and, when appropriate, the national regulatory authority.

19.15 Liability and Insurance

RVL subscribes to an insurance policy for insurance/indemnity in its terms and provisions, its legal liability as a Sponsor and a CI or delegate site for injuries caused to participating persons and arising out of this research performed strictly in accordance with the scientific protocol as well as with applicable law and professional standards.

19.16 Quality Assurance

The Sponsor will be responsible for implementing and maintain quality assurance and control system with written SOPs and as detailed in the Study Audit Plan to ensure the study is conducted and data generated, documented and reported in compliance with the protocol, GCP and regulatory requirements.

The Sponsor representative is responsible for securing agreement to ensure direct access to all trial related sites source documents and reports for the purpose of monitoring and auditing by the sponsor.

If such an audit occurs, the CI or delegate will give the auditor direct access to all relevant documents, and will allocate his time and the time of his staff to the auditor as may be required to discuss findings and any relevant issues.

In addition, regulatory agencies may conduct an inspection of this study. If such an inspection occurs, the CI or delegate will allow the inspector direct access to all source documents and other study documentation for source data checking and/or on-site audit inspection. The CI or delegate must allocate his time and the time of his staff to the inspector to discuss findings of any relevant issues.

19.17 Study Termination

Upon completion of the study, the following activities when applicable must be conducted by the Study Monitor in conjunction with the CI or delegate, as appropriate:

- Provision of all study data to the Sponsor
- Data clarifications and/or resolutions
- Review of site study records for completeness

In addition, the sponsor reserves the right to temporarily suspend or prematurely terminate this study for any reason (see Section 19.5).

20 DISCLOSURE OF DATA

20.1 Subject Confidentiality

The CI or delegate shall reassure subjects that their confidentiality will be maintained during all audits and inspections of the study site and documentation by third parties. A unique study number assigned to each subject at the start of the study, along with their initials, will be used to identify the subject on study documentation, on all study correspondence and in the study database. The CI or delegate will keep an identification code list and enrolment log which will list the full name of each subject alongside the subject number assigned and the date enrolled. This log will remain in the ISF at the study site. Subject names will not be supplied to the Sponsor.

20.2 Sponsor Confidentiality

RVL will use confidential information solely for the purpose of conducting this study, as specified in the contract between the CI or delegate and RVL.

20.3 Publication Policy

All information that has been provided by RVL and is unpublished is confidential and must remain the sole property of RVL. The CI or delegate will agree to use the information only for the purposes of carrying out this study and for no other purpose unless prior written permission from the Sponsor is obtained. The Sponsor has full ownership of the source documents completed as part of the study.

The CI or delegate may not submit the results of the study for publication without the prior consent of the Sponsor.

20.4 Study Documentation

20.4.1 Source Documents

The CI or delegate shall keep the ISFs and source documents until notified otherwise by the Sponsor.

RETROSCREEN VIROLOGY
CONQUERING VIRAL DISEASE

21 REFERENCES

1. Jones S, Evans K, McElwaine-Johnn H, Sharpe M, Oxford J, et al. (2009) DNA vaccination protects against an influenza challenge in a double-blind randomised placebo-controlled phase 1b clinical trial. Vaccine 27: 2506-2512.
2. DeVincenzo J, Lambkin-Williams R, Wilkinson T, Cehelsky J, Nochur S, et al. (2010) A randomized, double-blind, placebo-controlled study of an RNAi-based therapy directed against respiratory syncytial virus. Proc Natl Acad Sci U S A 107: 8800-8805.
3. Zaas AK, Chen M, Varkey J, Veldman T, Hero AO, 3rd, et al. (2009) Gene expression signatures diagnose influenza and other symptomatic respiratory viral infections in humans. Cell Host Microbe 6: 207-217.
4. Lillie PJ, Berthoud TK, Powell TJ, Lambe T, Mullarkey C, et al. (2012) Preliminary assessment of the efficacy of a T-cell-based influenza vaccine, MVA-NP+M1, in humans. Clin Infect Dis 55: 19-25.
5. Hershenson MB, Johnston SL (2006) Rhinovirus Infections. Am J Respir Crit Care Med 174: 1284-1285.
6. Kling S, Donninger H, Williams Z, Vermeulen J, Weinberg E, et al. (2005) Persistence of rhinovirus RNA after asthma exacerbation in children. Clin Exp Allergy 35: 672-678.
7. Hayden FG (2004) Rhinovirus and the lower respiratory tract. Rev Med Virol 14: 17-31.
8. Ong EL, Ellis ME, Webb AK, Neal KR, Dodd M, et al. (1989) Infective respiratory exacerbations in young adults with cystic fibrosis: role of viruses and atypical microorganisms. Thorax 44: 739-742.
9. Smyth M, Haas D, Friebe M (1995) The ups and downs for children with chronic illnesses. Manag Care Q 3: 91-95.
10. Wat D, Gelder C, Hibbitts S, Bowler I, Pierrepoint M, et al. (2008) Is there a role for influenza vaccination in cystic fibrosis? J Cyst Fibros 7: 85-88.
11. Gwaltney JM, Jr., Park J, Paul RA, Edelman DA, O'Connor RR, et al. (1996) Randomized controlled trial of clemastine fumarate for treatment of experimental rhinovirus colds. Clin Infect Dis 22: 656-662.
12. Flint SJASM (2009) Principles of Virology. Washington, DC: ASM Press.

22 APPENDICES

Appendix 1: Study Approved Contraceptive Methods 75

Appendix 2: Excluded Medical and Psychiatric Conditions 76

Appendix 3: RVL Symptom Diary Card 78

Appendix 1: Study Approved Contraceptive Methods

- Established use of oral, injected or implanted hormonal methods of contraception.
- Placement of an intrauterine device (IUD) or intrauterine system (IUS).
- Barrier methods of contraception: condom or occlusive cap (diaphragm or cervical/vault caps) with spermicidal foam/gel/film/cream/suppository.
- Male sterilisation (with the appropriate post-vasectomy documentation of the absence of sperm in the ejaculate). [For female subjects on the study, the vasectomised male partner should be the sole partner for that subject].
- True abstinence: When this is in line with the preferred and usual lifestyle of the subject. [Periodic abstinence (e.g., calendar, ovulation, symptothermal, post-ovulation methods) and withdrawal are not acceptable methods of contraception].

Appendix 2: Excluded Medical and Psychiatric Conditions

- Alcohol or drug dependency
- Allergic broncho-pulmonary aspergillosis
- Anaemia or other haematological condition
- Anorexia nervosa
- Any immunosuppressive condition whether acquired or congenital
- Asthma (The history of childhood asthma until and including age of 12 is acceptable)
- Autoimmune disease – history or evidence of
- Bipolar disorder
- Bronchiectasis
- Bronchitis
- Bulimia
- Chronic fatigue syndrome
- Chronic kidney disease
- Chronic liver disease
- Chronic lung condition of any aetiology
- COPD
- Cystic fibrosis or other congenital pulmonary condition
- Depression (exclusion for depression can be determined by CI)
- Diabetes mellitus- type 1 or type 2
- Disorders of bone, muscle or nervous system which may impact on clinical response to viral infection
- Emphysema
- Epistaxis - any clinically significant history of nose bleeds more than one per month.
- Hypo or hyperthyroidism
- Impaired immune responsiveness – known of any cause
- Inflammatory bowel disease
- Interstitial lung disease or pulmonary fibrosis
- Ischaemic heart disease
- Known IgA deficiency, immotile cilia syndrome, or Kartagener's syndrome
- Malignancy
- Morbid obesity
- Nasal surgery (any)within 6 months of inoculation
- Peptic ulcer disease (exclusion is dependent on CI's assessment of significance)
- Psychosis
- Pulmonary hypertension
- Reactive airway disease
- Significant chronic skin conditions
- Significant connective tissue or rheumatological disease
- Significant disease of upper airway, ears or eyes
- Sinus surgery (any) within 6 months of inoculum.

- Tuberculosis or any other significant history of respiratory infection
- Valvular heart disease or cardiac failure

Appendix 3: RVL Symptom Diary Card

Symptom Diary Card

Page 1 of 2

Subject Number ☐☐☐☐☐☐

Subject Initials ☐☐☐ F M L

Date ☐☐☐☐☐☐☐☐☐ D D M M M Y Y Y Y

RETROSCREEN VIROLOGY
CONQUERING VIRAL DISEASE

SYMPTOM DIARY CARD	Time of Day: ______ : ______ *(24 hour clock)*		Morning ☐ Afternoon ☐ Evening ☐ (mark with an X)	
**Level**	**0**	**1**	**2**	**3**
**Symptoms** <u>*Please report the symptoms you are experiencing at the moment*</u>	**I have NO symptoms**	**Just noticeable**	**It's clearly bothersome from time to time, but it doesn't stop me from participating in activities**	**It's quite bothersome most or all of the time, and it stops me from participating in activities**
**Runny Nose** *(mark with an X)*	☐ 0	☐ 1	☐ 2	☐ 3
**Stuffy Nose** *(mark with an X)*	☐ 0	☐ 1	☐ 2	☐ 3
**Sneezing** *(mark with an X)*	☐ 0	☐ 1	☐ 2	☐ 3
**Sore Throat** *(mark with an X)*	☐ 0	☐ 1	☐ 2	☐ 3
**Earache** *(mark with an X)*	☐ 0	☐ 1	☐ 2	☐ 3
**Malaise (tiredness)** *(mark with an X)*	☐ 0	☐ 1	☐ 2	☐ 3
**Cough** *(mark with an X)*	☐ 0	☐ 1	☐ 2	☐ 3
**Shortness of breath** *(mark with an X)*	☐ 0	☐ 1	☐ 2	☐ 3
**Headache** *(mark with an X)*	☐ 0	☐ 1	☐ 2	☐ 3
**Muscle and/or joint ache** *(mark with an X)*	☐ 0	☐ 1	☐ 2	☐ 3

Volunteer's Initials: ____________________ **Doctor's Initials:** ____________________

Date: ______ / ______ / ______
D D / M M M / Y Y Y Y

Time: ______ : ______
(24 hour clock)

Symptom Diary Card

Subject Number ☐☐☐☐☐☐

Subject Initials ☐☐☐ (F M L)

Date ☐☐☐☐☐☐☐☐☐ (D D M M M Y Y Y Y)

RETROS
CONQUE

SYMPTOM DIARY CARD	Time of Day: ______ : ______ *(24 hour clock)*		Morning ☐ Afternoon<br>Evening ☐ (mark with
**Symptoms**<br>*Please report the symptoms you are experiencing at the moment*	**Less Severe Symptom**	**More Severe Symptom**	
**Runny Nose**			*Please mark the scale with an ✗ to indicate*
**Stuffy Nose**			*Please mark the scale with an ✗ to indicate*
**Sneezing**			*Please mark the scale with an ✗ to indicate*
**Sore Throat**			*Please mark the scale with an ✗ to indicate*
**Earache**			*Please mark the scale with an ✗ to indicate*
**Malaise (tiredness)**			*Please mark the scale with an ✗ to indicate*
**Cough**			*Please mark the scale with an ✗ to indicate*
**Shortness of breath**			*Please mark the scale with an ✗ to indicate*
**Headache**			*Please mark the scale with an ✗ to indicate*
**Muscle and/or joint ache**			*Please mark the scale with an ✗ to indicate*

Volunteer's Initials: ____________ **Doctor's Initials:** ____________ **Date:** __ __ / __ __ __ / __ __ __ __ (*D D / M M M / Y Y Y Y*) **Time:** ______ *(24*
